# Supplementary material for: Wild and domesticated animal abundance is associated with greater late-Holocene alpine plant diversity
Source: Nat Commun. 2025 Apr 25;16:3924. doi: 10.1038/s41467-025-59028-2 (PMC12032255; doi:10.1038/s41467-025-59028-2)
Supplement: Supplementary file 1 — Supplementary Information [file 41467_2025_59028_MOESM1_ESM.pdf]

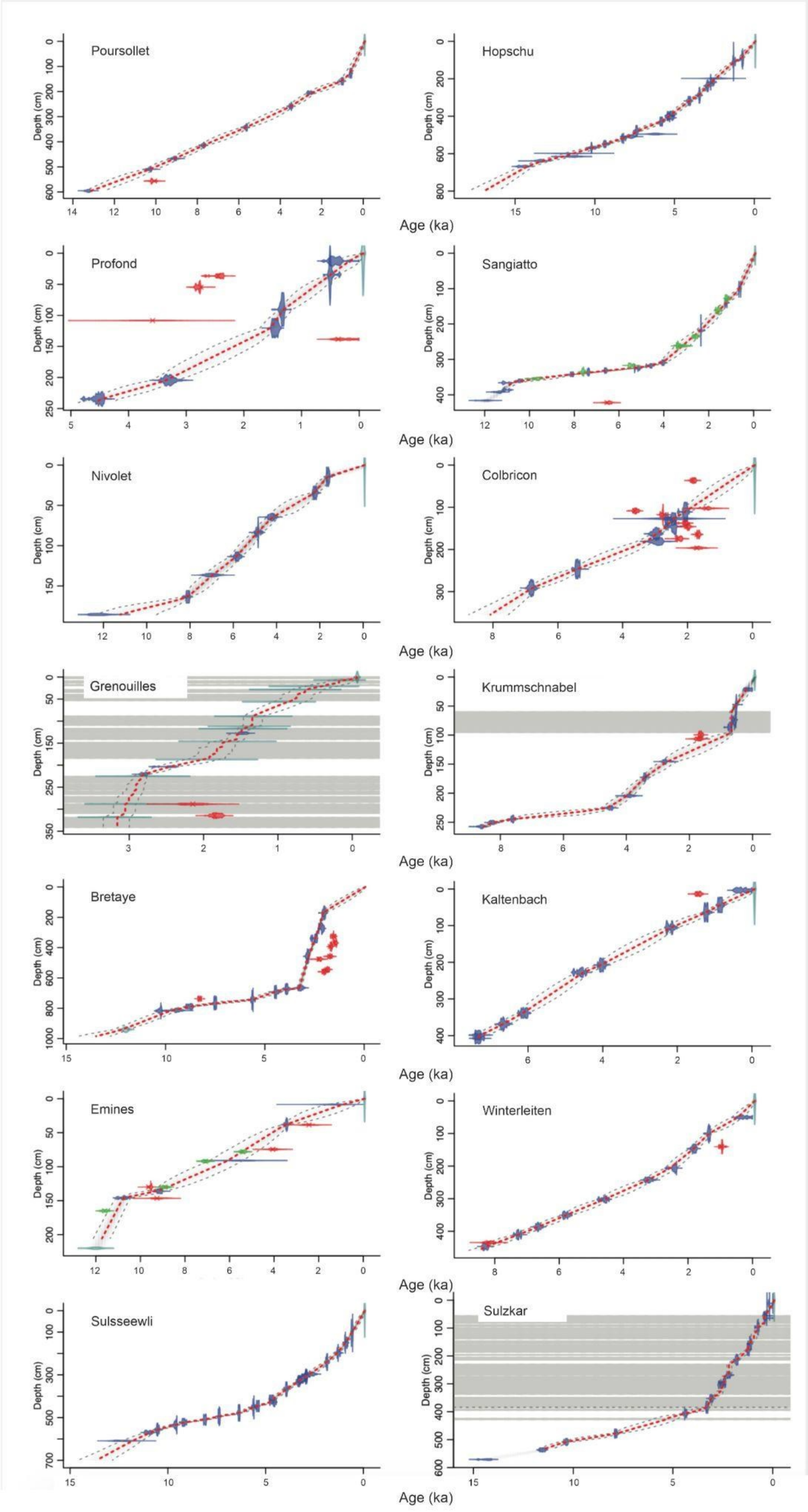

Supplementary Figure 1. *Age-depth models. Red points are outlier dates. Green points are dates based on regional pollen stratigraphy and can be used to check the age-depth model. Grey-shaded horizontal bands indicate rapid deposition events.*

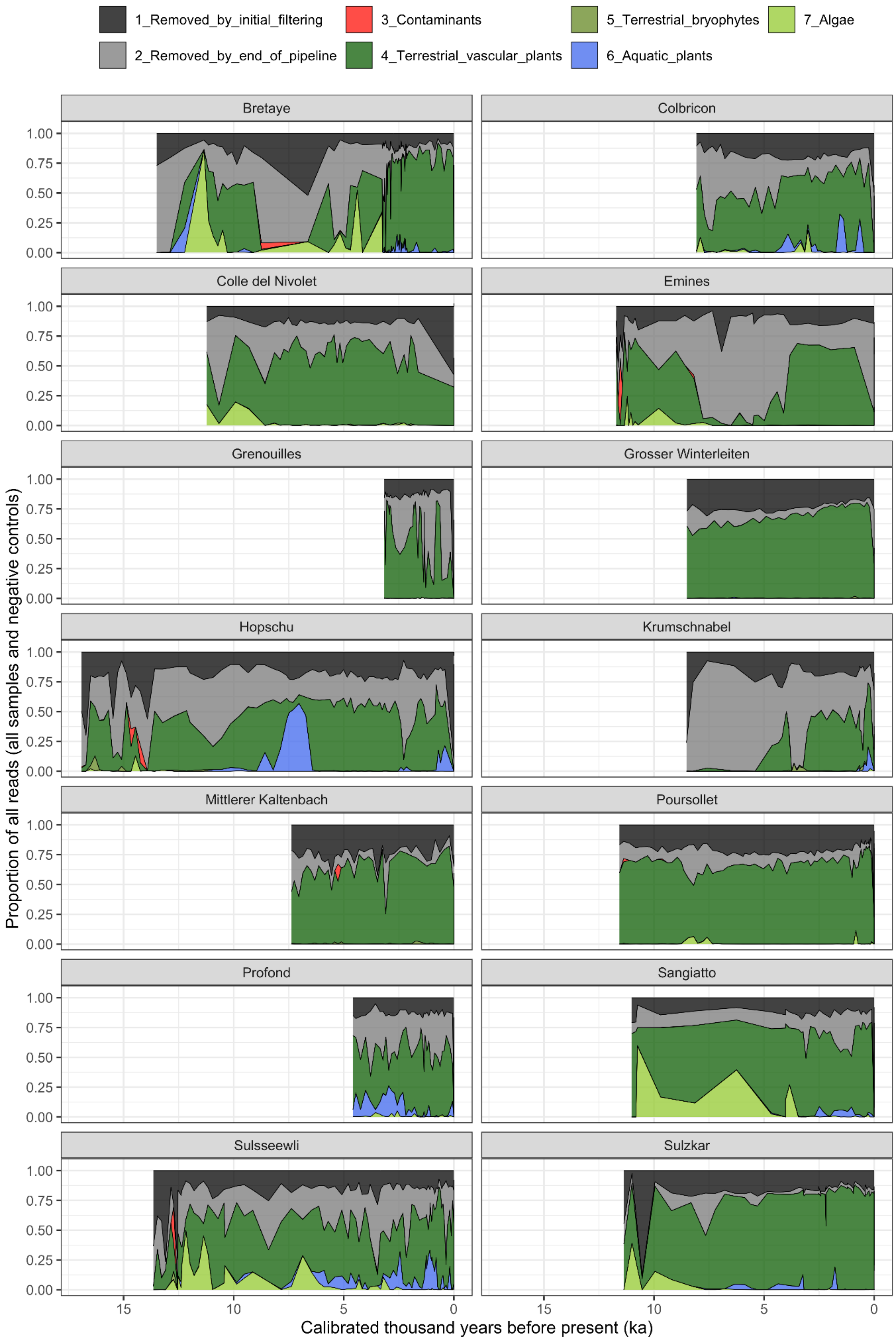

Supplementary Figure 2*. Higher-level classification of sequencing reads across all samples and negative controls in the data set. Negative controls have values near or at 0.*

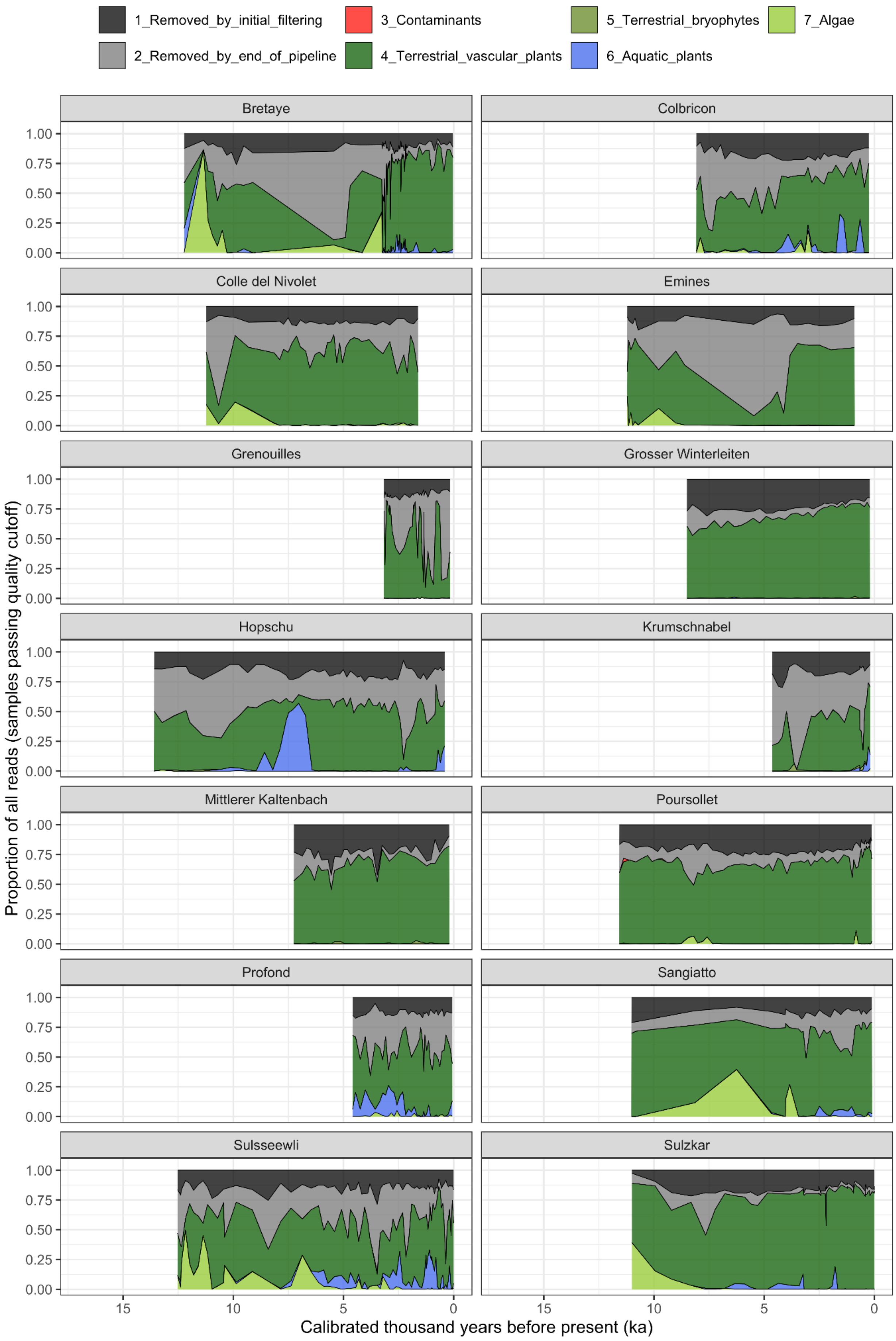

Supplementary Figure 3. *Higher-level classification of sequencing reads across samples that passed the MTQ/MAQ quality control criteria. These samples were used in all downstream analyses.*

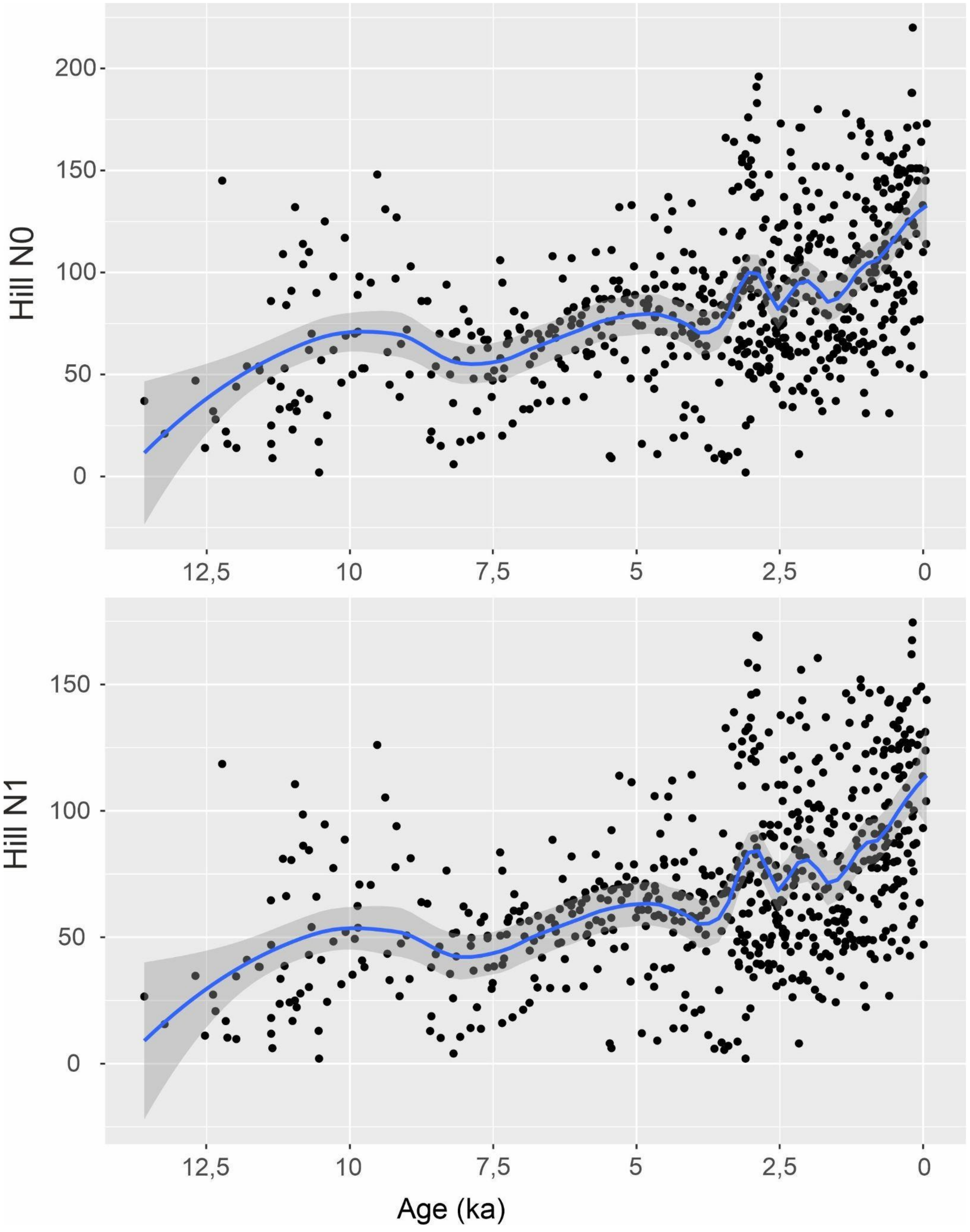

Supplementary Figure 4*. Total plant species diversity expressed as Hill N0 (richness) and Hill N1 of all lakes along the Holocene using Loess (Span=0.2)(n = 649 samples).*

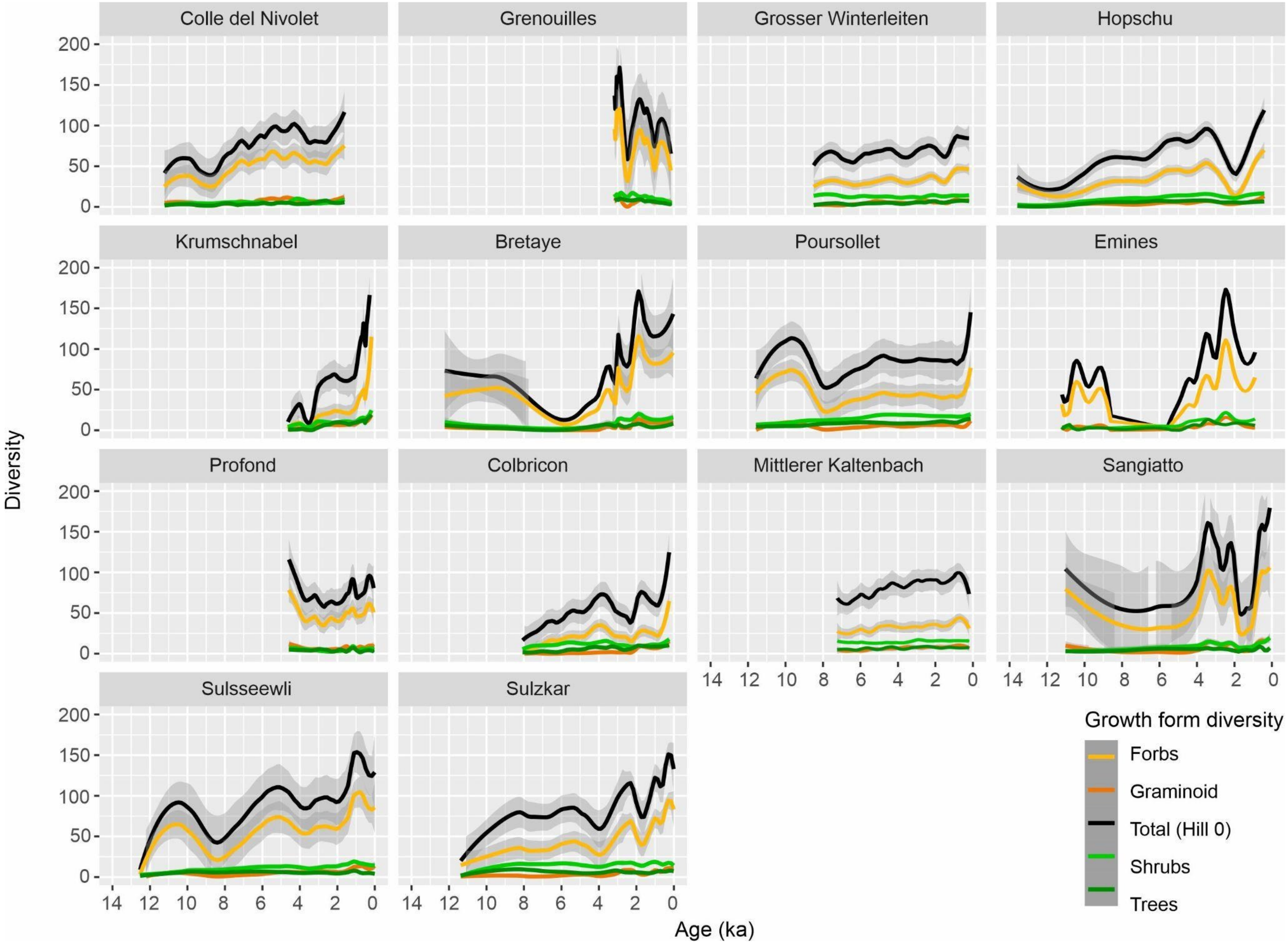

***Supplementary Figure 5**. Total plant species diversity and diversity for each growth form group measured as Hill N0 (richness). Smoothed curves using Span=0.25.*

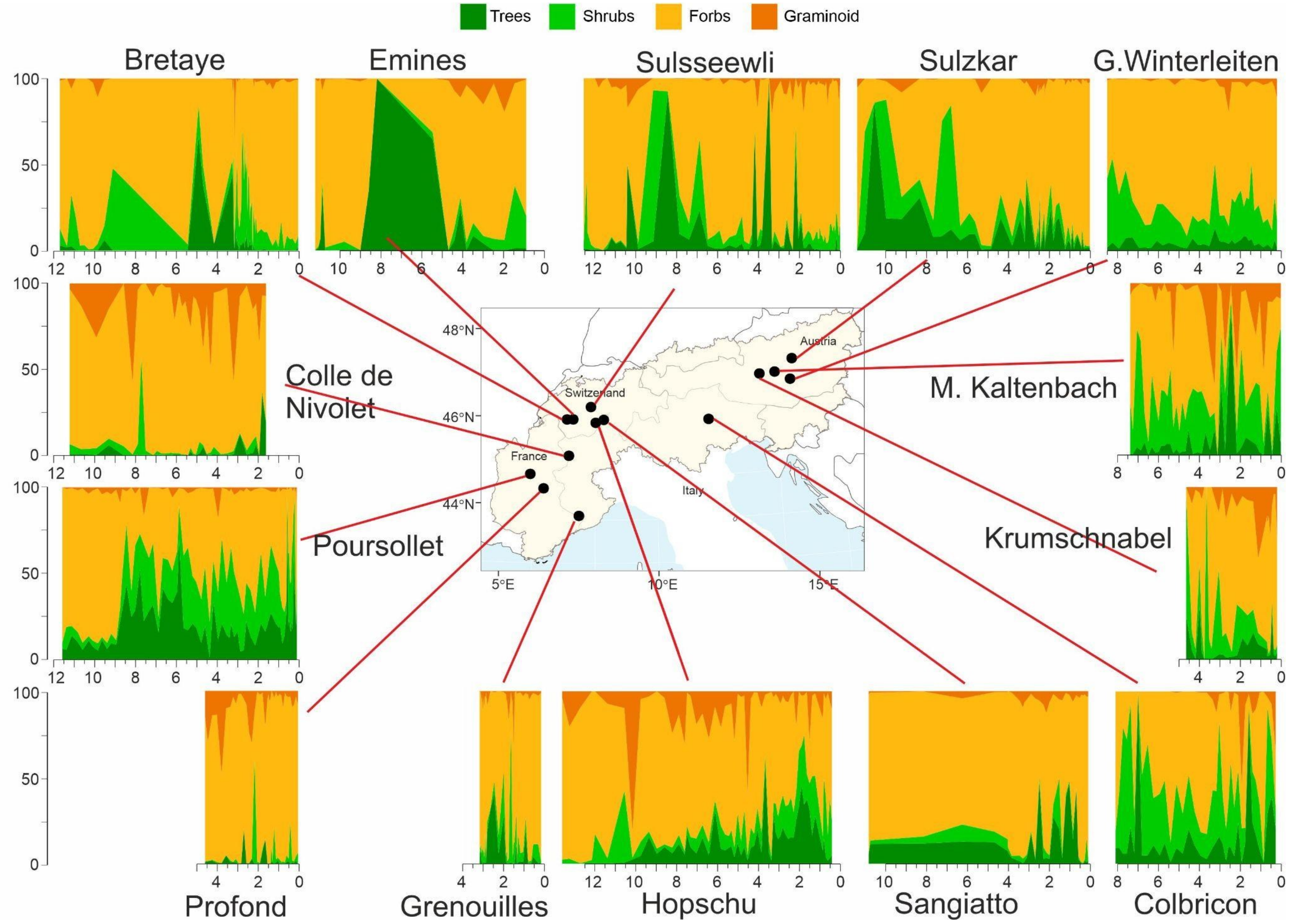

Supplementary Figure 6*. Relative abundance (RAI)* of growth *forms for the last 12 ka. Map is drawn from data from Natural Earth.*

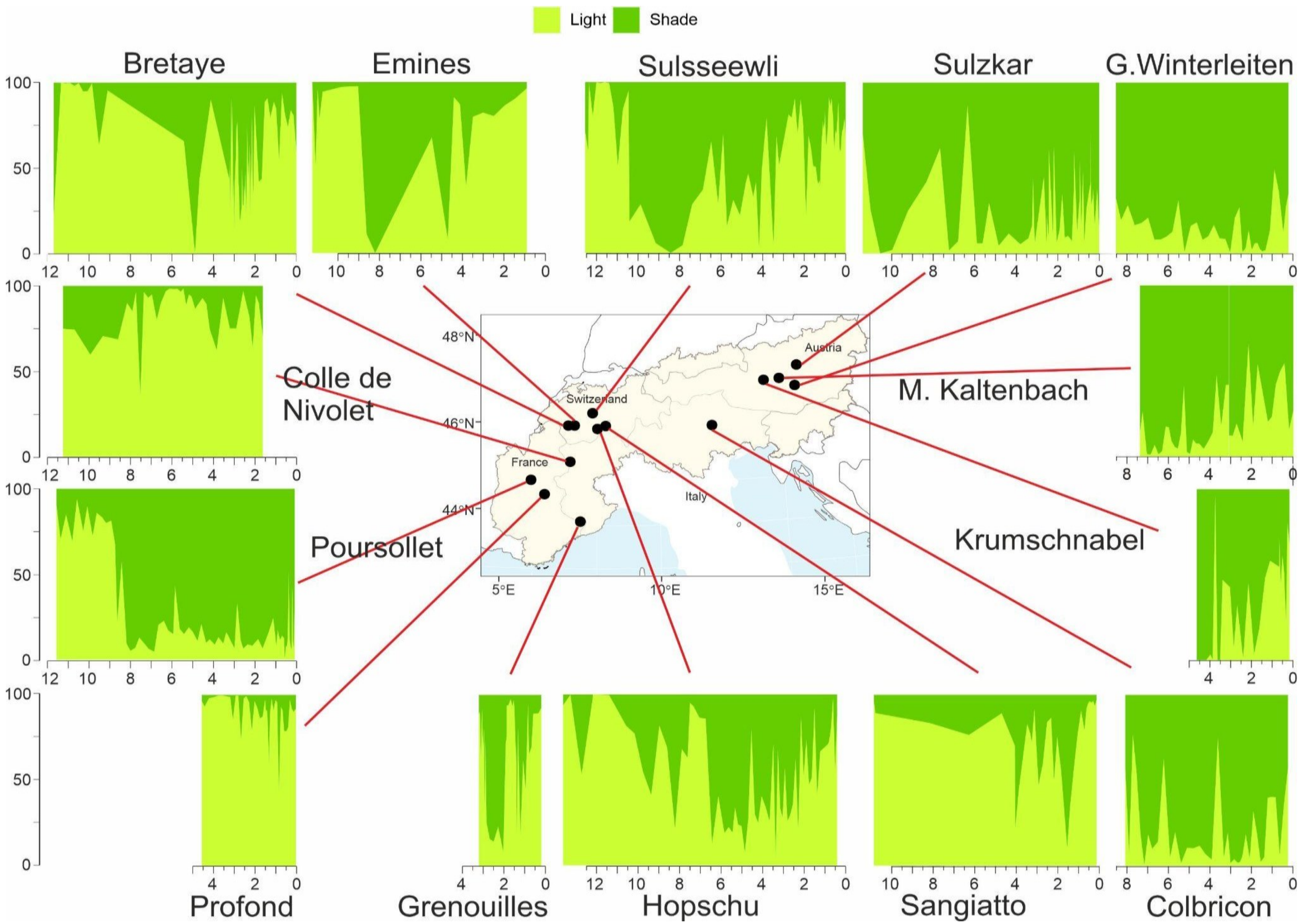

***Supplementary Figure 7**. Relative abundance (RAI) of light and shade indicator taxa for the last 12 ka. Map is drawn from data from Natural Earth.*

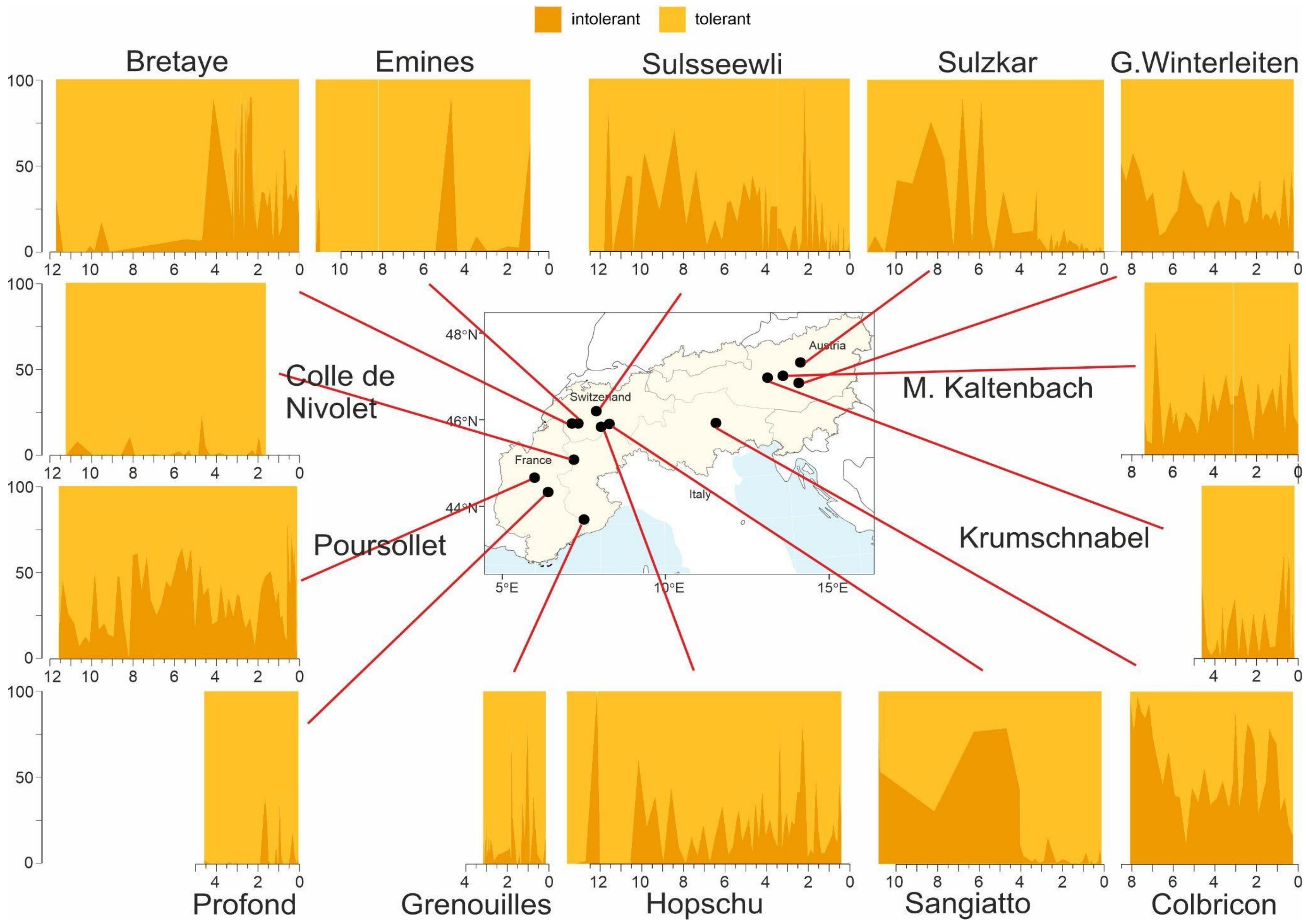

Supplementary Figure 8. *Relative abundance (RAI) of grazing tolerant taxa for the last 12 ka. Map is drawn from data from Natural Earth.*

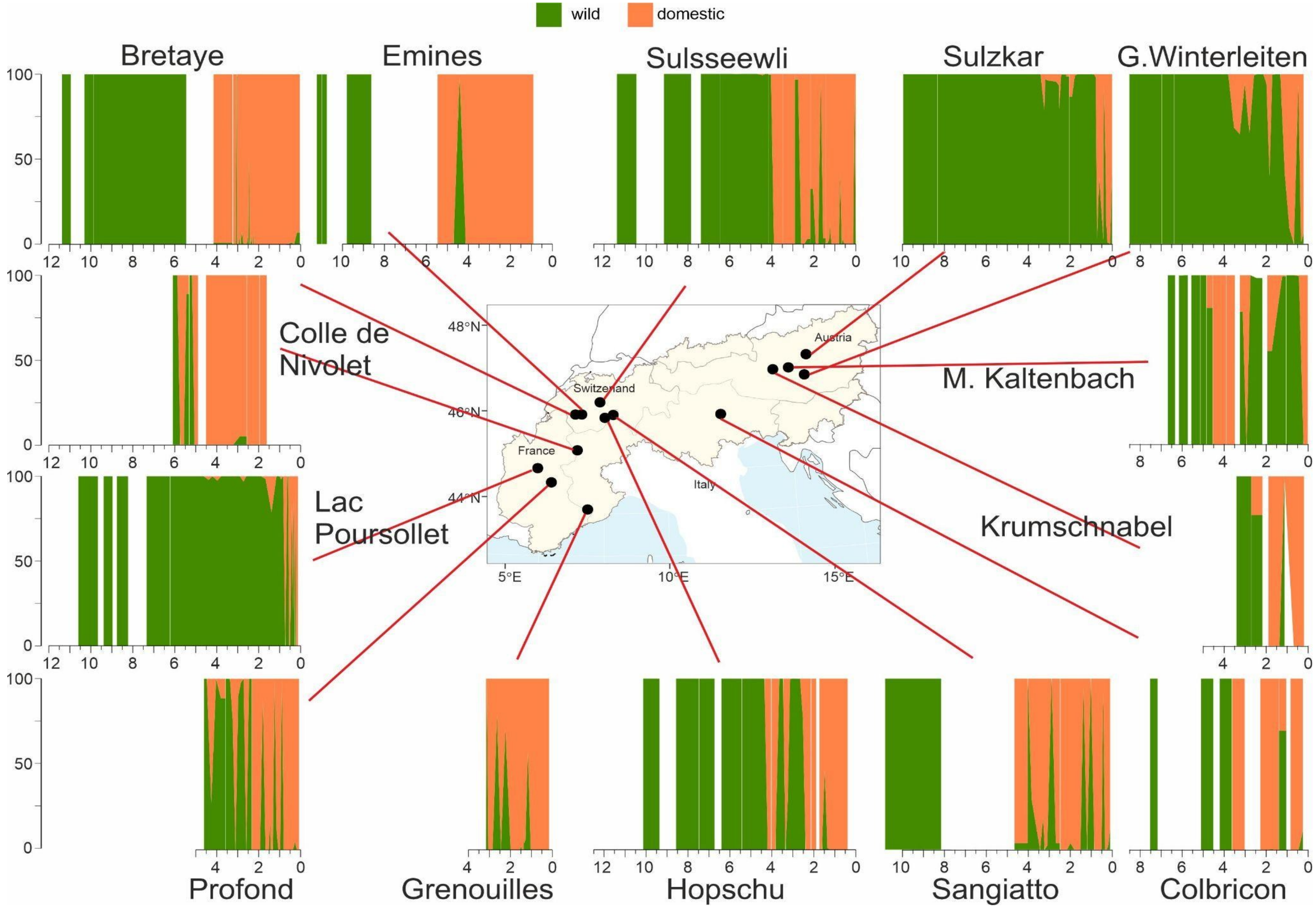

***Supplementary Figure 9**. Wild and domesticated mammal relative abundance (RAI) for the last 12 ka. Map is drawn from data from Natural Earth.*

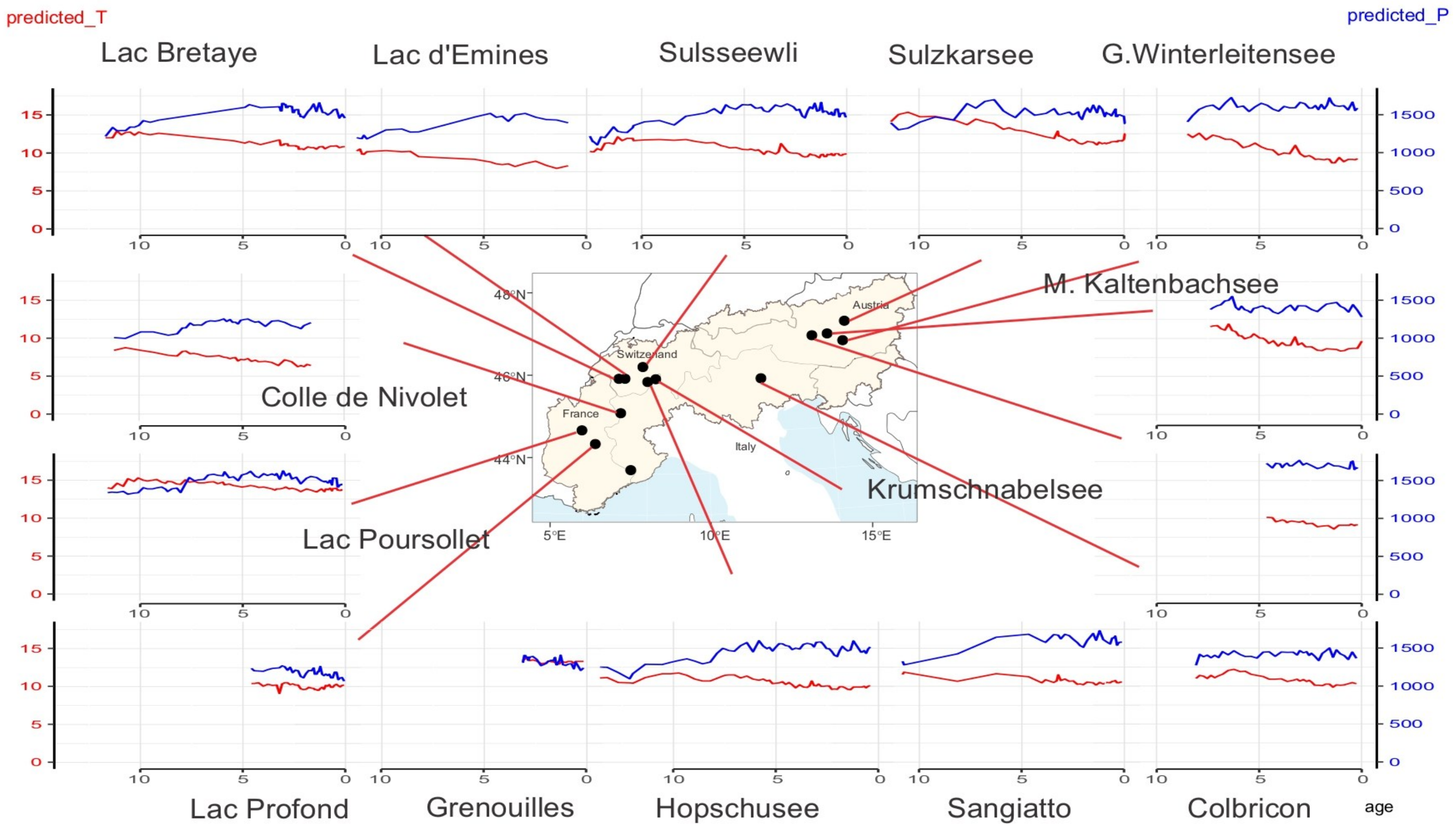

__Supplementary Figure 10__. Temperature (red) and precipitation (blue) for the last 10 ka at each of the 14 lake sites. Map is drawn from data from Natural Earth.

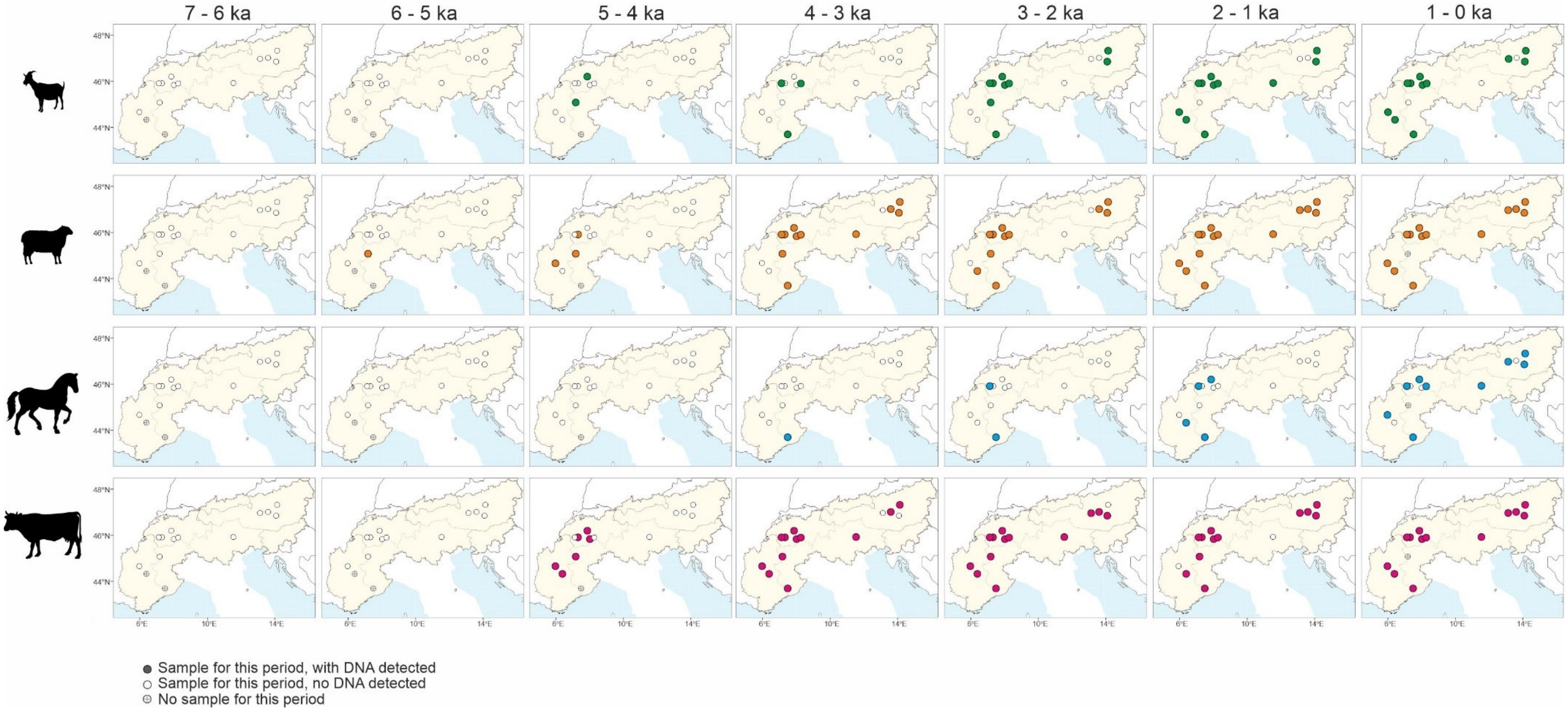

Supplementary Figure 11. *Domestic presence of herbivores in all lakes for the last 7 ka. Silouhettes comes from Phylopic (https://www.phylopic.org/). Map is drawn from data from Natural Earth.*

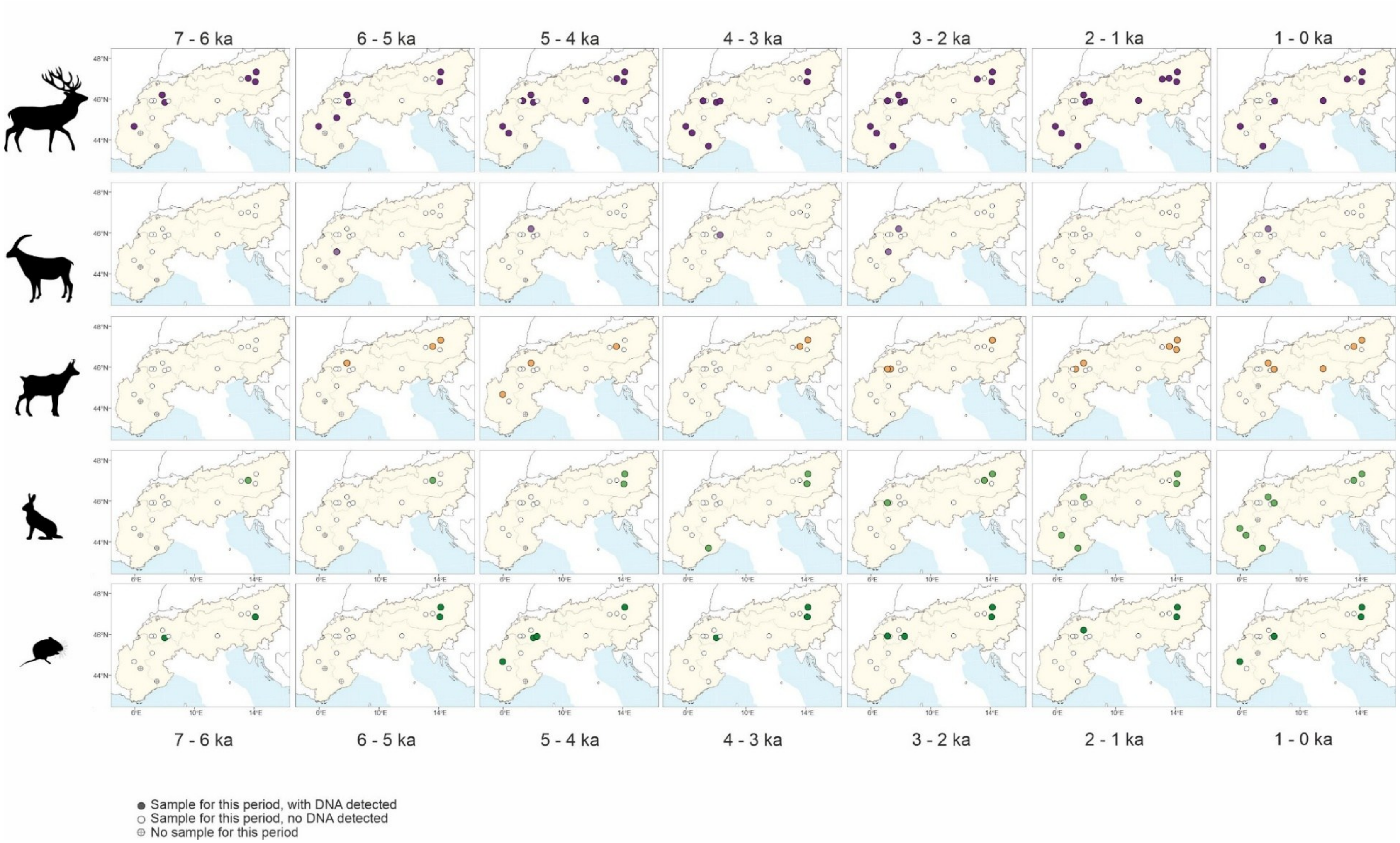

Supplementary Figure 12*. Wild presence of herbivores in all lakes for the last 7 ka. Silouhettes comes from Phylopic (https://www.phylopic.org/). Map is drawn from data from Natural Earth.*

Supplementary Table 1. Location of the 14 lakes

Lake	Code	Age (cal ka yr BP)	Altitude (m)	Country	Upper potential treeline (msm)	Elevational distance to treeline	Current vegetation belt of the lake catchment	Latitude	Longitude	Previous publications
Krumschnabel	EG22	8.5	1989	Austria	1900	+89	subalpine to lower alpine	47.26272	13.54914	
Grosser Winterleiten	EG23	8.5	1845	Austria	1950	-105	high montane to upper alpine	47.089653	14.566058	(1)
Mittlerer Kaltenbach	EG25	7.3	1912	Austria	1800	+116	subalpine to upper alpine	47.28193	14.06432	
Grenouilles	EG28	3.1	1999	France	2350	-351	lower to upper subalpine belt	44.09823	7.48358	
Profond	EG30	4.5	2480	France	2250	+230	alpine belt	44.724734	6.343380	
Colle del Nivolet	EG31	11.2	2529	Italy	2200	+329	alpine	45.48622	7.14548	
Sulsseewli	EG33	13.6	1921	Switzerland	2000	-79	subalpine to alpine belt	46.6175	7.86388	(2)
Emines	EG34	11.7	2288	Switzerland	2100	+188	alpine belt	46.32888	7.275833	(3)
Poursollet	EG35	10.8	1661	France	2150	-489	lower subalpine to alpine belt	45.0512	5.89852	
Colbricon	EG38	8	1862	Italy	2200	-338	subalpine	46.28182	11.7649	(4)
Hopschu	EG39	16.9	2017	Switzerland	2250	-233	subalpine to alpine belt	46.25222	8.02305	(5–7)

Bretaye	EG40	12	1780	Switzerland	2100	-320	lower subalpine to alpine belt	46.3261	7.07194	(8)
Sangiatto	EG41	11	1980	Italy	2200	-220	subalpine to alpine belt	46.31916	8.290167	(9)
Sulzkar	EG42	11,3	1450	Austria	1750	-300	upper montane to subalpine	47.559755	14.680075	

Supplementary Table 2. Total samples and controls for both the trnL p6-loop and 16S data sets

Lake	Code	Samples	Failed Quality Control	Passed Quality Control	Controls	Negative Controls	Positive Controls	Total
Krumschnabel	EG22	37	9	28	8	7	1	**45**
Grosser Winterleiten	EG23	37	0	37	8	7	1	**45**
Mittlerer Kaltenbach	EG25	39	7	32	8	5	1	**45**
Grenouilles	EG28	40	0	40	8	7	1	**48**
Profond	EG30	40	0	40	8	17	1	**48**
Colle del Nivolet	EG31	38	1	37	8	7	1	**46**
Sulseewlii	EG33	80	8	72	16	14	2	**96**
Emines	EG34	35	15	20	8	7	1	**43**
Poursollet	EG35	68	0	68	16	14	2	**84**
Colbricon	EG38	38	0	38	8	7	1	**46**
Hopschu	EG39	76	16	60	16	14	2	**92**
Bretaye	EG40	80	7	73	16	14	2	**96**
Sangiatto	EG41	34	2	32	8	7	1	**42**
Sulzkar	EG42	62	2	60	6	5	1	**68**

**Total**		**704**	**67**	**637**	**140**	**122**	**18**	**844**

Supplementary Table 3 Plant sequences after filtering

	Taxa
Original sequences	1215
After sequence check	747
Contaminant taxa	25
Algae	19
Aquatic	17
Cyanobacteria	1
Fern	29
Forb	421
Graminoid	49
Horsetail	5
Liverwort	8
Moss	85
Shrub	44
Tree	38

Supplementary Note 1. Lake descriptions

Switzerland

Hopschu lake is a small subalpine lake located in the Swiss canton of Valais (2017 m a.s.l, 46.151111 N; 8.012222 E), a region shaped by glaciers in the Southern Central Alps (10). It is located in the west of Simplon Pass (2005 m a.s.l), which is the lowest major mountain pass in the area that connects the Pennine Alps with the Lepontine Alps in Italy. The pond is 165 m long and 78 m wide, and presents some inflows and no visible outflow. It is characterized by a continental climate with hot and dry summers and cold winters. This lake is found in the limit of the subalpine vegetation stage, above the timberline. The catchment is dominated by *Rhododendron ferrugineum* shrubs and other Ericaceae (*Vaccinium* spp*, Arctostaphylos uva-ursi, Calluna vulgaris* and *Empetrum nigrum* spp). The littoral of the pond includes a high proportion of grasses and Cyperaceae, while some *Potamogeton* spp and *Carex* spp are found in the lake. Scattered *P. cembra* and *Larix* trees can be observed around the catchment. Welten (1982) studied the palaeoecology of this lake based on pollen.

Emines is located in the West Central Alps, close to Sanetsch Pass (Valais, Switzerland) and 2.45 km east of the Glacier de Tsanfleuron. Situated in the alpine belt (2288 m a.s.l, 46.617639° N, 7.864028° E), the surrounding vegetation is characterized by *Seslerion* grass communities (*Sesleria caerulea, Leontopodium alpinum* or *Carex sempervirens*) (3). The pond has a surface area of 0.5 ha and a water depth of 1.5 m. It has no visible inflow or outflow. The catchment lies on calcareous bedrock, surrounded by bare rock and scree slopes, together with meadows and a scattered spruce (*Picea abies*) forest on the eastern side.

Sulsseewli is a small lake situated in the Bernese Alps (North central Alps). It is located in the subalpine vegetation belt (1921 m a.s.l.; 46.617639° N, 7.864028° E), below the treeline. The catchment is mostly dominated by grasses with a small stand of spruce (*Picea abies*) on the northern slope. Sulsseewli has two water inlets and a subaquatic outlet, with a maximum water depth of ca 7.5 m.

Bretaye is a subalpine lake located in the West Central Swiss Alps (Vaud, Switzerland, 1780 m a.s.l, 46.32623 N; 7.072089 E). It has a surface area of 4 ha and a minor inflow (8). Located in the subalpine vegetation belt, the lake catchment is dominated by spruce (*Picea abies*) and green Alder (*Alnus viridis*), with scarce presence of larch (*Larix decidua*) stone pine (*Pinus cembra*) and sycamore (*Acer pseudoplatanus*). The lake is surrounded by pastures and some shrubs of *Juniperus communis* and *Rhododendron hirsutum*.

Austria

Krummschnabel is located in the subalpine belt near Obertauern, in the province of Salzburg (Eastern Central Alps; 1989 m a.s.l; 47.26272 N; 13.54914 E). The rather small catchment is dominated by grasses (*Avenella flexuosa ssp. flexuosa, Nardus stricta*) and Ericaceae shrubs (*Rhododendron ferrugineum* and *Vaccinium myrtillus*) with scattered Mountain Pine (*Pinus mugo*). Juncaceae such as *Juncus filiformis* and *Luzula alpina* abound around the lake. The aquatic vegetation consists of *Ranunculus confervoides* and *Sparganium angustifolium* with some rare presence of *Potamogeton* sp. The catchment is currently used as a cattle pasture. The lake is horseshoe-shaped at 75 m in width at the widest point by 150 m long and has a water depth of 4.7 m. There is no obvious inlet, rather there are boggy (with *Sphagnum spp.*) and spring vegetation (with *Drepanocladus sp.*) sites, and a small, probably seasonally dry outlet.

Grosser Winterleiten is a high montane / subalpine lake located in Eastern Central Alps (Zirbizkogel, Austria; 1,845 m a.s.l; 47.089653 N; 14.566058 E), lying to the North East of Zirbizkogel (2,396 m a.s.l), the highest point of the Seetaler Alpen. The lake is 200 m at its widest point and 300 m in length. The lake has one main inlet and one outlet. The closer catchment is dominated by mixed forests of Swiss Stone pine (*Pinus cembra*), Larch (*Larix decidua*) and Spruce (*Picea abies*). Some *Alnus alnobetula* ssp*. alnobetula* and *Salix* spp. are also present in the moisture regions (1). Further up the catchment area extends to the alpine belt.

The understory is represented by *Juniperus communis, Rhododendron ferrugineum*, *Vaccinium myrtillus* and *Calluna vulgaris*. *Carex rostrata* and *Carex nigra* predominate along the littoral. The west part of the lake is surrounded by a wetland of *Sphagnum capillifolium, Sphagnum subsecundum* and *Sphagnum warnstorfii.* The northern slope of Grosser Winterleiten is dominated by meadows of *Deschampsia caespitosa* with some *Veratrum album ssp. album* and *Rumex alpestris* that indicate grazing activities occurred in the past. This lake is no longer used for intensive domesticate pasturing, since the overtake of the area by the Austrian armed forces.

Mittlerer Kaltenbach is a sub-alpine lake in the Eastern Central Alps, (province of Styria Austria, 47.28195 N, 14.06368 E), it is the middle of three lakes with the name Kaltenbach. The lake is 250 m long and 150 m wide, and has one inlet and one outlet. The closer catchment is dominated by subalpine shrubs (*Alnus alnobetula*, *Pinus mugo*) and grassland dominated by *Nardus stricta* as well as shrubs of Ericaceae. *Sphagnum spp.* is growing directly at the shore of the lake. Scattered moist grassland patches with *Carex nigra* are also frequently present.

Sulzkar is a subalpine lake located in the Eastern Central Alps (Styria, Austria; 47.5598° N; 14.6804° E). It is the only lake within the Gesäuse National Park. The bedrock is dominated by various types of limestones, covered by moraine material and soils with high clay content. The lake has a seasonally dry inlet and no outlet. The water level of the lake is varying seasonally. There is only one small creek that carries meltwater in spring, which is usually dry, and only has water during heavy rains. Cattle and horses slightly pasture the area around the lake. One side of the lake is characterized by an open montane forest with rocks and screes with spruce (*Picea abies*), stone pine (*Pnus cembra*) and maple (*Acer pseudoplatanus*), whereas the other side has characteristics of an open subalpine pasture. The plant species diversity is very high because of the structured landscape and very high precipitation in this area. As for water plants, a yet unidentified pondweed dominates.

Italy

Superior Colbricon lake is located in the Lagorai mountain range, inside the Paneveggio-Pale di San Martino Natural Park (Trentino, Southern Central Alps)(1922 m, 46.28182 N; 11.7649 E). The bedrock is composed of quartziferous porphyry and sandstone. The lake is situated in the subalpine vegetation belt, characterised by a mixed forest of Scots pine (*Pinus cembra*), mountain pine (*Pinus mugo* ssp*. mugo*), spruce (*Picea abies*) and larch (*Larix decidua*). The understorey is characterized by shrubs such as *Rhododendron ferrugineum, Vaccinium myrtillus, Vaccinium uliginosum* and *Juniperus sibirica*. The predominant grasses are Cyperaceae and *Calamagrostis* sp. (11). The climate is wet and cold, with a mean annual precipitation of 1157 mm and a mean annual temperature of 2.7 °C at Rolle Pass weather station (2002 m a.s.l.), approximately 3 km from Colbricon lake (12). The lake has a maximum depth of 12 m and an area of 0.024 km^2. The lakes lie on peat soil, over a bedrock substratum of quartziferous porphyry, and are surrounded by a *Sphagnum* and sedge vegetation (11).

Inferiore del Sangiatto is a subalpine pond located in the Southern Central Alps (1980 m a.s.l.; 46.31916 N, 8.290167 E), inside the Veglia-Devero Natural Park. The lake has a maximum water depth of 3 m and a 1 ha surface area, with one minor surface inflow and no surface outflow. The catchment is surrounded by an open larch (*Larix decidua*) forest, with single *Pinus cembra* trees, Ericaceae, *Juniperus* shrubs and alpine meadows used as pastures. The predominant aquatics are *Sparganium angustifolium, Potamogeton alpinum* and *Ranunculus trichophyllus* (Bionda et al 2013). Today, the treeline is located at c. 2250 m a.s.l. and the timberline at c. 2050 m asl, placing Sangiatto just below the treeline ecotone. Geologically, the area around the lake belongs to the Lebendun nappe and consists of calc-schists and mica-schists (13). The climate is mild and wet, with a mean annual temperature of c. 2.0 °C and 1520 mm of annual precipitation. The mean temperature of the coldest (January) and warmest month (July) are c. -6.0 °C and 11 °C respectively (adjusted to the elevation of Lago Sangiatto with a lapse rate of 6 °C/1000 m from the nearby meteorological station at Alpe Devero; Arpa Piemonte, 2019).

Colle del Nivolet inferior lake is located in the Gran Paradiso National Park (GPNP) (45.48629 N; 7.14548 E, 2529 m), in the Graian Alps (Western Italian Alps). It forms the watershed between the Aosta Valley and Piedmont. The mean elevation of its peaks is 3000 m and the highest peak is the Gran Paradiso (4061 m). The lake is close to Rifugio Savoia and is located above the treeline. The surrounding vegetation consists mainly of pastures and grasses. The local geology is dominated by acidic gneiss that forms the Gran Paradiso massif. Colle del Nivolet is a highly-protected, closed hydrological basin between about 2500 and 2700 m a.s.l. This area,

covered with snow from November to June, is characterized by a complex environment of alpine pastures, oligotrophic lakes, peat bogs, rock outcrops, and meandering streams.

France

Grenouilles (44° 6’ 6.64” N; 7° 28’ 32.7” E, 1999 m a.s.l.) is located in Mercantour National Park (France), at the southern end of the Alpine arc. The maximum lake depth is 3 m. There are several cattle herds and ovine flocks in the area surrounding and bordering the lake. This lake is near Mount Bégo, where 40,000 rock engravings dating back to the early Bronze Age can be found, being the largest open-air engraving collection in the world.

Profond (44.724734° 6.343380° 2480 m a.s.l.) is situated on the western edge of the Ecrins (France). One of six lakes situated between 2400 and 2560 m in the commune of Orcières in the Champsaur region, Lac Profond is 140 m long in a north-south direction with an inflow to the north and an outflow to the south. It is 60 m wide. The geological context comprises sandstone flysch (Aiguilles d'Arves flysch, Champsaur sandstone and Annot sandstone) that is Priabonian in age (Lower Oligocene). The lake is 400 m above the current treeline. The wider area has a long history of pastoral activity (demonstrated by combined archaeological and palynological research). The area has traditionally seen a combination of bovine and ovine grazing.

Poursollet (45.0512 N; 5.89852 E; 1661m) is located in the Taillefer massif close to Livet-et-Gavet on the western edge of the French Alps (Auvergne-Rhône-Alpes region). The lake is ovoid in shape with a small protrusion on its northern end, has a maximum length of 212 m, average diameter of 100 m, and maximum depth of around 7.2 m. It is located at the bottom of a small basin, bordered to the west by the Pas des Escaliers (1694 m) and to the east by the le Taillefer mountain (2857 m). The lake is bordered by a small residential hamlet. This area comprises a variety of habitats for numerous animal and plant species thanks to the diverse environments. The surrounding forest has a variety of species; the bogs shelter numerous insect and amphibian species. The current timberline is around 2000 m, i.e. 340 m above the lake. The lowest lake amongst the sites studied here has a long history of pastoral activity in its environs. Currently, the pastures are made of cows and the pastoral pressure is very low.

Supplementary Note 2. Bibliography

1. Zetter, S., Garcés-Pastor, S., Lammers, Y., Brown, A. G., Walsh, K., Goslar, T., ... & Greve Alsos, I. (2024). SedaDNA shows that transhumance of domestic herbivores has enhanced plant diversity over the Holocene in the Eastern European Alps. The Holocene, 09596836241307304. .

2. Garcés-Pastor S, Coissac E, Lavergne S, Schwörer C, Theurillat J-P, Heintzman PD, et al. High resolution ancient sedimentary DNA shows that alpine plant diversity is associated with human land use and climate change. Nat Commun. 2022 Nov 4;13(1):6559.

3. Berthel N, Schwörer C, Tinner W. Impact of Holocene climate changes on alpine and treeline vegetation at Sanetsch Pass, Bernese Alps, Switzerland. Rev Palaeobot Palynol. 2012 Apr;174:91–100.

4. Belle S, Musazzi S, Tõnno I, Poska A, Leys B, Lami A. Long-term effects of climate change on carbon flows through benthic secondary production in small lakes. Freshwater Biology. 2018 Jun;63(6):530–8.

5. van der Knaap WO, van Leeuwen JFN, Fankhauser A, Ammann B. Palynostratigraphy of the last centuries in Switzerland based on 23 lake and mire deposits: chronostratigraphic pollen markers, regional patterns, and local histories. Rev Palaeobot Palynol. 2000 Jan;108(1–2):85–142.

6. Ammann B, van der Knaap WO, Lang G, Gaillard M-J, Kaltenrieder P, Rösch M, et al. The potential of stomata analysis in conifers to estimate presence of conifer trees: examples from the Alps. Veg Hist Archaeobot. 2014 May;23(3):249–64.

7. Welten M. Vegetationsgeschichtliche Untersuchungen in den westlichen Schweizer Alpen: Bern-Wallis (Textheft+ Diagrammheft). 1982;

8. Thöle L, Schwörer C, Colombaroli D, Gobet E, Kaltenrieder P, van Leeuwen J, et al. Reconstruction of Holocene vegetation dynamics at Lac de Bretaye, a high-mountain lake in the Swiss Alps. The Holocene. 2016 Mar;26(3):380–96.

9. van Vugt L, Garcés-Pastor S, Gobet E, Brechbühl S, Knetge A, Lammers Y, et al. Pollen, macrofossils and sedaDNA reveal climate and land use impacts on Holocene mountain vegetation of the Lepontine Alps, Italy. Quat Sci Rev. 2022 Nov;296:107749.

10. Dielforder A, Hetzel R. The deglaciation history of the Simplon region (southern Swiss Alps) constrained by 10Be exposure dating of ice-molded bedrock surfaces. Quat Sci Rev. 2014 Jan;84:26–38.

11. Festi F, Prosser F. La flora del Parco Naturale Paneveggio Pale di S. Martino: atlante corologico e repertorio delle segnalazioni. 2000;

12. Castagneri D, Vacchiano G, Lingua E, Motta R. Analysis of intraspecific competition in two subalpine Norway spruce (Picea abies (L.) Karst.) stands in Paneveggio (Trento, Italy). Forest Ecology and Management. 2008 Mar;255(3–4):651–9.

13. Piana F, Fioraso G, Irace A, Mosca P, d'Atri A, Barale L, et al. Geology of Piemonte region (NW Italy, Alps–Apennines interference zone). J Maps. 2017 Nov 30;13(2):395–405.
