# Supplementary material for: Wild and domesticated animal abundance is associated with greater late-Holocene alpine plant diversity
Source: Nat Commun. 2025 Apr 25;16:3924. doi: 10.1038/s41467-025-59028-2 (PMC12032255; doi:10.1038/s41467-025-59028-2)
Supplement: Supplementary file 2 — Description of Additional Supplementary Files [file 41467_2025_59028_MOESM2_ESM.pdf]

Description of Additional Supplementary Files

Supplementary Data 1. Radiocarbon dates

Supplementary Data 2. Samples names, depths, and ages of all sedaDNA samples

Supplementary Data 3. TrnL p6loop plant sequences

Supplementary Data 4. 16S mammal sequences

Supplementary Data 5. 16S sequences in FASTA format

Supplementary Data 6. 16S haplotypes of domesticated mammals

Supplementary Data 7. Excluded samples of domesticated mammals

Supplementary Data 8. Plants found in the controls (wtRep)

Supplementary Data 9. Mammals found in the controls (wtRep)

Supplementary Data 10. Plant sample information

Supplementary Data 11. Table with all data for the Figures

Supplementary Code 1. Statistical Analyses
