# Supplementary material for: Wild and domesticated animal abundance is associated with greater late-Holocene alpine plant diversity
Source: Nat Commun. 2025 Apr 25;16:3924. doi: 10.1038/s41467-025-59028-2 (PMC12032255; doi:10.1038/s41467-025-59028-2)
Supplement: Supplementary file 4 — Supplementary Code 1 [file 41467_2025_59028_MOESM4_ESM.zip › NCOMMS-24-59826A Supp Code 1.docx]

**Supplementary Code**

- Supplementary Code 1: Statistical Analyses

**Supplementary Code 1**

plantas_RAI <- read.csv("Metadata_samples_All_Alps.26.01.2024.csv", sep=";", dec =".", header=T)

**# removing all 0s in dataset**

plantas_RAI <- subset(plantas_RAI,Elevation>0 & predicted_T>0 & predicted_P>0 & DNA_Hill_N1!=0)

**# removing controls, bad quality and superficial samples**

plantas_RAI <- plantas_RAI %>% filter(bad_quality==F & sample_type=="sample")

**# Centering predictors by lake**

plantas_RAI_na <- subset(plantas_RAI, !is.na(predicted_T))

plantas_RAI_na$cent_T <- residuals(lm(predicted_T~lake_name, data=plantas_RAI_na)) # taking residuals to center by lakes

plantas_RAI_na$cent_P <- residuals(lm(predicted_P~lake_name, data=plantas_RAI_na))

plantas_RAI_na$lake_T <- fitted(lm(predicted_T~lake_name, data=plantas_RAI_na)) # taking fitted to have mean by lakes

plantas_RAI_na$lake_P <- fitted(lm(predicted_P~lake_name, data=plantas_RAI_na))

plantas_RAI_na2 <- subset(plantas_RAI_na, !is.na(Bos_taurus))

**##### transforming proportions to have a better spread**

plantas_RAI_na2$Bos_taurus_l <- log(plantas_RAI_na2$Bos_taurus+0.001)

plantas_RAI_na2$Ovis_l <- log(plantas_RAI_na2$Ovis+0.001)

plantas_RAI_na2$Capra_l <- log(plantas_RAI_na2$Capra+0.001)

plantas_RAI_na2$Ibex_l <- log(plantas_RAI_na2$Capra_ibex+0.001)

plantas_RAI_na2$Rupicapra_l <- log(plantas_RAI_na2$Rupicapra+0.001)

plantas_RAI_na2$Cervus_l <- log(plantas_RAI_na2$Cervus_elaphus+0.001)

plantas_RAI_na2$Equus_caballus_l <- log(plantas_RAI_na2$Equus_caballus+0.001)

plantas_RAI_na2$Equus_asinus_l <- log(plantas_RAI_na2$Equus_asinus+0.001)

plantas_RAI_na2$Myodes_glareolus_l <- log(plantas_RAI_na2$Myodes_glareolus+0.001)

plantas_RAI_na2$Glis_glis_l <- log(plantas_RAI_na2$Glis_glis+0.001)

plantas_RAI_na2$Castor_fiber_l <- log(plantas_RAI_na2$Castor_fiber+0.001)

plantas_RAI_na2$Lepus_timidus_l <- log(plantas_RAI_na2$Lepus_timidus+0.001)

plantas_RAI_na2$Talpa_europaea_l <- log(plantas_RAI_na2$Talpa_europaea+0.001)

plantas_RAI_na2$Neomys_fodiens_l <- log(plantas_RAI_na2$Neomys_fodiens+0.001)

plantas_RAI_na2$Meles_meles_l <- log(plantas_RAI_na2$Meles_meles+0.001)

plantas_RAI_na2$Arvicola_amphibius_l <- log(plantas_RAI_na2$Arvicola_amphibius+0.001)

plantas_RAI_na2$Sciurus_vulgaris_l <- log(plantas_RAI_na2$Sciurus_vulgaris+0.001)

plantas_RAI_na2$Sorex_l <- log(plantas_RAI_na2$Sorex+0.001)

plantas_RAI_na2$Martes_martes_l <- log(plantas_RAI_na2$Martes_martes+0.001)

plantas_RAI_na2$Camelus_l <- log(plantas_RAI_na2$Camelus+0.001)

plantas_RAI_na2$Ursus_arctos_l <- log(plantas_RAI_na2$Ursus_arctos+0.001)

plantas_RAI_na2$Vulpes_vulpes_l <- log(plantas_RAI_na2$Vulpes_vulpes+0.001)

**### with Hill N0 : Hill_0_richness**

gam.m4 <- gamm(Hill_0_richness ~ s(lake_T) + s(cent_T) + s(lake_P) + s(cent_P) + s(Ovis_l) + s(Bos_taurus_l) + s(Capra_l) + s(Ibex_l) + s(Rupicapra_l) + s(Cervus_l) + s(Equus_caballus_l) + s(Myodes_glareolus_l) + s(Lepus_timidus_l) +s(Talpa_europaea_l) +s(Neomys_fodiens_l) + s(Ursus_arctos_l) , random=list(lake_name=~1), data = plantas_RAI_na2)

plot(gam.m4$gam, residuals=T, pages=1, pch=19)

gam.lmer4 <- lmer(Hill_0_richness ~ lake_T + cent_T + lake_P + cent_P + Ovis_l + Bos_taurus_l + Capra_l + Ibex_l + Rupicapra_l + Cervus_l +Equus_caballus_l + Myodes_glareolus_l + Lepus_timidus_l + Talpa_europaea_l + Neomys_fodiens_l + Ursus_arctos_l + (1|lake_name), data = plantas_RAI_na2)

summary(gam.lmer4)

REML criterion at convergence: 5964.3

Scaled residuals:

Min 1Q Median 3Q Max

-3.0989 -0.5662 -0.0108 0.5632 5.5411

Random effects:

Groups Name Variance Std.Dev.

lake_name (Intercept) 192.6 13.88

Residual 708.7 26.62

Number of obs: 637, groups: lake_name, 14

Fixed effects:

Estimate Std. Error t value

(Intercept) 456.52745 67.20955 6.793

lake_T 2.90770 2.39572 1.214

cent_T -5.99867 1.67579 -3.580

lake_P -0.02201 0.02832 -0.777

cent_P 0.02848 0.01315 2.166

Ovis_l 8.89154 2.24775 3.956

Bos_taurus_l 26.30491 2.18114 12.060

Capra_l 1.78029 2.44081 0.729

Ibex_l 10.47804 3.03829 3.449

Rupicapra_l 12.33892 2.63156 4.689

Cervus_l 6.77423 1.78333 3.799

Equus_caballus_l 2.65418 2.59040 1.025

Myodes_glareolus_l -7.37494 3.10551 -2.375

Lepus_timidus_l -0.52019 3.00421 -0.173

Talpa_europaea_l -6.31198 3.73413 -1.690

Neomys_fodiens_l -2.24341 3.32285 -0.675

Ursus_arctos_l 2.90714 3.14018 0.926


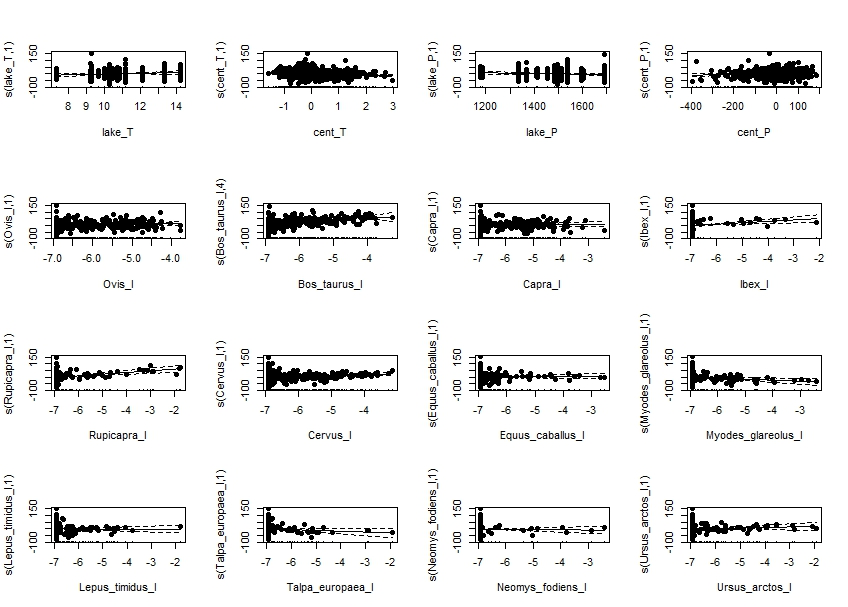


summary(gam.m4$gam)

Family: gaussian

Link function: identity

Formula:

Hill_0_richness ~ s(lake_T) + s(cent_T) + s(lake_P) + s(cent_P) +

s(Ovis_l) + s(Bos_taurus_l) + s(Capra_l) + s(Ibex_l) + s(Rupicapra_l) +

s(Cervus_l) + s(Equus_caballus_l) + s(Myodes_glareolus_l) +

s(Lepus_timidus_l) + s(Talpa_europaea_l) + s(Neomys_fodiens_l) +

s(Ursus_arctos_l)

Parametric coefficients:

Estimate Std. Error t value Pr(>|t|)

(Intercept) 88.089 3.261 27.01 <2e-16 ***

---

Signif. codes: 0 ‘***’ 0.001 ‘**’ 0.01 ‘*’ 0.05 ‘.’ 0.1 ‘ ’ 1

Approximate significance of smooth terms:

edf Ref.df F p-value

s(lake_T) 1.000 1.000 1.992 0.158616

s(cent_T) 1.000 1.000 8.289 0.004128 **

s(lake_P) 1.000 1.000 1.301 0.254396

s(cent_P) 1.000 1.000 4.570 0.032926 *

s(Ovis_l) 1.000 1.000 6.703 0.009854 **

s(Bos_taurus_l) 3.998 3.998 45.026 < 2e-16 ***

s(Capra_l) 1.000 1.000 1.187 0.276368

s(Ibex_l) 1.000 1.000 13.296 0.000289 ***

s(Rupicapra_l) 1.000 1.000 24.900 1.34e-06 ***

s(Cervus_l) 1.000 1.000 17.510 3.32e-05 ***

s(Equus_caballus_l) 1.000 1.000 0.651 0.419931

s(Myodes_glareolus_l) 1.000 1.000 6.989 0.008409 **

s(Lepus_timidus_l) 1.000 1.000 0.004 0.950608

s(Talpa_europaea_l) 1.000 1.000 2.733 0.098773 .

s(Neomys_fodiens_l) 1.000 1.000 0.418 0.518335

s(Ursus_arctos_l) 1.000 1.000 1.738 0.187913

---

Signif. codes: 0 ‘***’ 0.001 ‘**’ 0.01 ‘*’ 0.05 ‘.’ 0.1 ‘ ’ 1

R-sq.(adj) = 0.449

Scale est. = 667.35 n = 637

**### Diversity within functional groups**

**Trees**

gam.trees.m1 <- gamm(Trees_F_richness ~ s(lake_T) + s(cent_T) + s(lake_P) + s(cent_P) + s(Ovis_l) + s(Bos_taurus_l) + s(Capra_l) + s(Ibex_l) + s(Rupicapra_l) + s(Cervus_l) + s(Equus_caballus_l) + s(Myodes_glareolus_l) + s(Lepus_timidus_l) + s(Talpa_europaea_l) + s(Neomys_fodiens_l) + s(Ursus_arctos_l), random=list(lake_name=~1), data = plantas_RAI_na2)

plot(gam.trees.m1$gam, residuals=T, pages=1, pch=19)

gam.trees.lmer <- lmer(Trees_F_richness ~ lake_T + cent_T + lake_P + cent_P + Ovis_l + Bos_taurus_l + Capra_l + Ibex_l + Rupicapra_l + Cervus_l + Equus_caballus_l + Myodes_glareolus_l + Lepus_timidus_l + Talpa_europaea_l + Neomys_fodiens_l + Ursus_arctos_l + (1|lake_name), data = plantas_RAI_na2)

summary(gam.trees.lmer)

REML criterion at convergence: 3033.9

Scaled residuals:

Min 1Q Median 3Q Max

-2.9321 -0.6091 -0.0143 0.5367 4.5734

Random effects:

Groups Name Variance Std.Dev.

lake_name (Intercept) 1.687 1.299

Residual 6.278 2.506

Number of obs: 637, groups: lake_name, 14

Fixed effects:

Estimate Std. Error t value

(Intercept) 0.822788 6.312078 0.130

lake_T 0.483757 0.224317 2.157

cent_T -0.685659 0.157723 -4.347

lake_P 0.001885 0.002652 0.711

cent_P 0.003653 0.001238 2.952

Ovis_l 0.367522 0.211548 1.737

Bos_taurus_l 0.448453 0.205277 2.185

Capra_l 0.003441 0.229723 0.015

Ibex_l -0.236943 0.285963 -0.829

Rupicapra_l -0.313411 0.247673 -1.265

Cervus_l 0.476018 0.167836 2.836

Equus_caballus_l -0.003984 0.243807 -0.016

Myodes_glareolus_l 0.325767 0.292290 1.115

Lepus_timidus_l 0.016195 0.282753 0.057

Talpa_europaea_l -0.633129 0.351460 -1.801

Neomys_fodiens_l 0.293764 0.312750 0.939

Ursus_arctos_l -0.304007 0.295543 -1.029


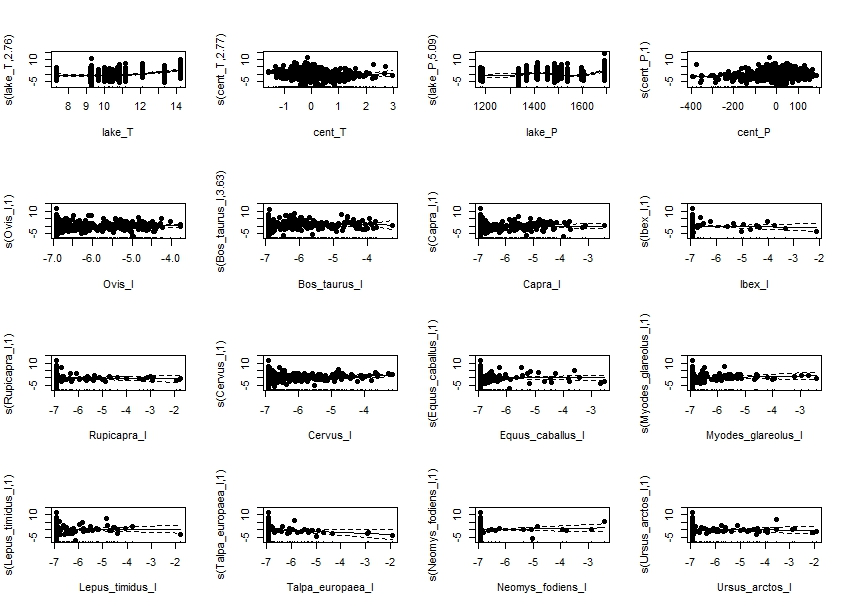


summary(gam.trees.m1$gam)

Family: gaussian

Link function: identity

Formula:

Trees_F_richness ~ s(lake_T) + s(cent_T) + s(lake_P) + s(cent_P) +

s(Ovis_l) + s(Bos_taurus_l) + s(Capra_l) + s(Ibex_l) + s(Rupicapra_l) +

s(Cervus_l) + s(Equus_caballus_l) + s(Myodes_glareolus_l) +

s(Lepus_timidus_l) + s(Talpa_europaea_l) + s(Neomys_fodiens_l) +

s(Ursus_arctos_l)

Parametric coefficients:

Estimate Std. Error t value Pr(>|t|)

(Intercept) 6.08007 0.09646 63.03 <2e-16 ***

---

Signif. codes: 0 ‘***’ 0.001 ‘**’ 0.01 ‘*’ 0.05 ‘.’ 0.1 ‘ ’ 1

Approximate significance of smooth terms:

edf Ref.df F p-value

s(lake_T) 2.759 2.759 35.876 < 2e-16 ***

s(cent_T) 2.766 2.766 7.893 5.32e-05 ***

s(lake_P) 5.088 5.088 9.827 < 2e-16 ***

s(cent_P) 1.000 1.000 9.788 0.001841 **

s(Ovis_l) 1.000 1.000 0.221 0.638110

s(Bos_taurus_l) 3.633 3.633 5.306 0.000448 ***

s(Capra_l) 1.000 1.000 0.010 0.920233

s(Ibex_l) 1.000 1.000 0.411 0.521574

s(Rupicapra_l) 1.000 1.000 0.377 0.539554

s(Cervus_l) 1.000 1.000 10.541 0.001231 **

s(Equus_caballus_l) 1.000 1.000 0.001 0.970252

s(Myodes_glareolus_l) 1.000 1.000 1.346 0.246468

s(Lepus_timidus_l) 1.000 1.000 0.086 0.769215

s(Talpa_europaea_l) 1.000 1.000 3.666 0.056012 .

s(Neomys_fodiens_l) 1.000 1.000 0.788 0.375068

s(Ursus_arctos_l) 1.000 1.000 0.050 0.822508

---

Signif. codes: 0 ‘***’ 0.001 ‘**’ 0.01 ‘*’ 0.05 ‘.’ 0.1 ‘ ’ 1

R-sq.(adj) = 0.337

Scale est. = 5.9169 n = 637

**Shrubs**

gam.shrubs.m1 <- gamm(Shrubs_F_richness ~ lake_T + cent_T + lake_P + cent_P + s(Ovis_l) + s(Bos_taurus_l) + s(Capra_l) + s(Ibex_l) + s(Rupicapra_l) + s(Cervus_l) + s(Equus_caballus_l) + s(Myodes_glareolus_l) + s(Lepus_timidus_l) + s(Talpa_europaea_l) + s(Neomys_fodiens_l) + s(Ursus_arctos_l), random=list(lake_name=~1), data = plantas_RAI_na2)

plot(gam.shrubs.m1$gam, residuals=T, pages=1, pch=19)

gam.shrubs.lmer <- lmer(Shrubs_F_richness ~ lake_T + cent_T + lake_P + cent_P + Ovis_l + Bos_taurus_l + Capra_l + Ibex_l + Rupicapra_l + Cervus_l + Equus_caballus_l + Myodes_glareolus_l + Lepus_timidus_l + Talpa_europaea_l + Neomys_fodiens_l + Ursus_arctos_l + (1|lake_name), data = plantas_RAI_na2)

summary(gam.shrubs.lmer)

REML criterion at convergence: 3325.4

Scaled residuals:

Min 1Q Median 3Q Max

-4.1981 -0.6122 -0.0089 0.6484 5.2186

Random effects:

Groups Name Variance Std.Dev.

lake_name (Intercept) 6.698 2.588

Residual 9.894 3.146

Number of obs: 637, groups: lake_name, 14

Fixed effects:

Estimate Std. Error t value

(Intercept) 11.189628 9.928054 1.127

lake_T 0.816180 0.434929 1.877

cent_T -0.755693 0.198306 -3.811

lake_P 0.010204 0.005117 1.994

cent_P 0.014499 0.001554 9.328

Ovis_l 1.214443 0.266519 4.557

Bos_taurus_l 2.017595 0.258760 7.797

Capra_l 0.239080 0.289089 0.827

Ibex_l -0.397220 0.359335 -1.105

Rupicapra_l 1.030132 0.311846 3.303

Cervus_l 0.750706 0.211697 3.546

Equus_caballus_l -0.488599 0.306426 -1.595

Myodes_glareolus_l -0.752781 0.367240 -2.050

Lepus_timidus_l -0.039076 0.355489 -0.110

Talpa_europaea_l -0.414647 0.441279 -0.940

Neomys_fodiens_l -0.131012 0.392724 -0.334

Ursus_arctos_l 0.512541 0.372076 1.378


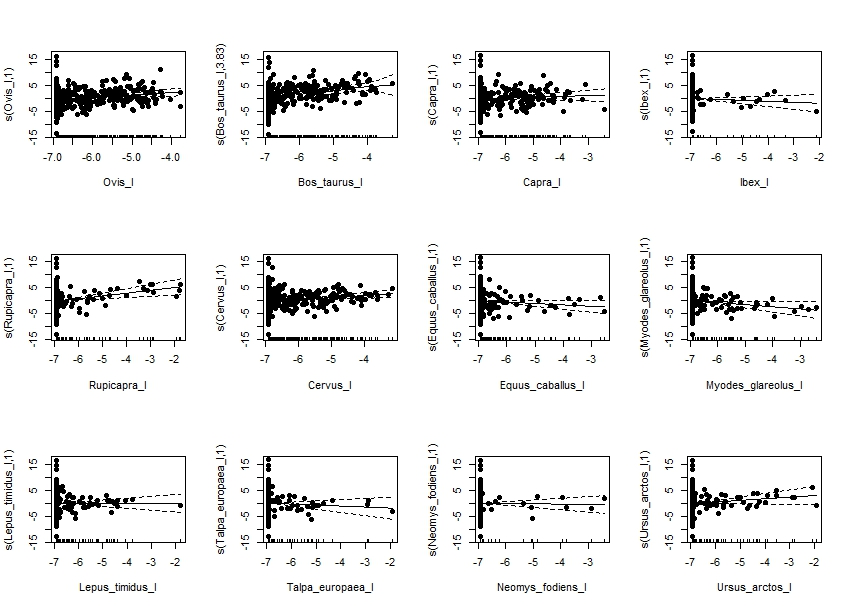


summary(gam.shrubs.m1$gam)

Family: gaussian

Link function: identity

Formula:

Shrubs_F_richness ~ lake_T + cent_T + lake_P + cent_P + s(Ovis_l) +

s(Bos_taurus_l) + s(Capra_l) + s(Ibex_l) + s(Rupicapra_l) +

s(Cervus_l) + s(Equus_caballus_l) + s(Myodes_glareolus_l) +

s(Lepus_timidus_l) + s(Talpa_europaea_l) + s(Neomys_fodiens_l) +

s(Ursus_arctos_l)

Parametric coefficients:

Estimate Std. Error t value Pr(>|t|)

(Intercept) -11.061668 6.934513 -1.595 0.11119

lake_T 0.809455 0.384453 2.105 0.03565 *

cent_T -0.632324 0.196269 -3.222 0.00134 **

lake_P 0.009754 0.004528 2.154 0.03161 *

cent_P 0.014350 0.001522 9.427 < 2e-16 ***

---

Signif. codes: 0 ‘***’ 0.001 ‘**’ 0.01 ‘*’ 0.05 ‘.’ 0.1 ‘ ’ 1

Approximate significance of smooth terms:

edf Ref.df F p-value

s(Ovis_l) 1.000 1.000 11.505 0.000739 ***

s(Bos_taurus_l) 3.828 3.828 21.590 < 2e-16 ***

s(Capra_l) 1.000 1.000 1.247 0.264620

s(Ibex_l) 1.000 1.000 1.120 0.290325

s(Rupicapra_l) 1.000 1.000 12.221 0.000507 ***

s(Cervus_l) 1.000 1.000 14.975 0.000121 ***

s(Equus_caballus_l) 1.000 1.000 3.238 0.072456 .

s(Myodes_glareolus_l) 1.000 1.000 5.106 0.024187 *

s(Lepus_timidus_l) 1.000 1.000 0.000 0.995534

s(Talpa_europaea_l) 1.000 1.000 0.787 0.375251

s(Neomys_fodiens_l) 1.000 1.000 0.091 0.763285

s(Ursus_arctos_l) 1.000 1.000 2.827 0.093203 .

---

Signif. codes: 0 ‘***’ 0.001 ‘**’ 0.01 ‘*’ 0.05 ‘.’ 0.1 ‘ ’ 1

R-sq.(adj) = 0.43

Scale est. = 9.3891 n = 637

**Forbs**

gam.forbs.m1 <- gamm(Forbs_F_richness ~ lake_T + cent_T + lake_P + cent_P + s(Ovis_l) + s(Bos_taurus_l) + s(Capra_l)+ s(Ibex_l) + s(Rupicapra_l) + s(Cervus_l) + s(Equus_caballus_l) + s(Myodes_glareolus_l) + s(Lepus_timidus_l) + s(Talpa_europaea_l) + s(Neomys_fodiens_l) + s(Ursus_arctos_l), random=list(lake_name=~1), data = plantas_RAI_na2)

plot(gam.forbs.m1$gam, residuals=T, pages=1, pch=19)

gam.forbs.lmer <- lmer(Forbs_F_richness ~ lake_T + cent_T + lake_P + cent_P + Ovis_l + Bos_taurus_l + Capra_l + Ibex_l + Rupicapra_l + Cervus_l + Equus_caballus_l + Myodes_glareolus_l + Lepus_timidus_l + Talpa_europaea_l + Neomys_fodiens_l + Ursus_arctos_l + (1|lake_name), data = plantas_RAI_na2)

summary(gam.forbs.lmer)

Linear mixed model fit by REML ['lmerMod']

Formula: Forbs_F_richness ~ lake_T + cent_T + lake_P + cent_P + Ovis_l +

Bos_taurus_l + Capra_l + Ibex_l + Rupicapra_l + Cervus_l +

Equus_caballus_l + Myodes_glareolus_l + Lepus_timidus_l +

Talpa_europaea_l + Neomys_fodiens_l + Ursus_arctos_l + (1 | lake_name)

Data: plantas_RAI_na2

REML criterion at convergence: 5465.8

Scaled residuals:

Min 1Q Median 3Q Max

-3.0145 -0.5424 -0.0431 0.5405 4.9362

Random effects:

Groups Name Variance Std.Dev.

lake_name (Intercept) 138.1 11.75

Residual 314.6 17.74

Number of obs: 637, groups: lake_name, 14

Fixed effects:

Estimate Std. Error t value

(Intercept) 362.524575 49.740973 7.288

lake_T 2.705712 1.994918 1.356

cent_T -2.785317 1.117659 -2.492

lake_P -0.044381 0.023511 -1.888

cent_P 0.006140 0.008764 0.701

Ovis_l 6.475148 1.500992 4.314

Bos_taurus_l 18.977946 1.456986 13.025

Capra_l 1.226304 1.628780 0.753

Ibex_l 8.646477 2.025660 4.268

Rupicapra_l 9.508668 1.756643 5.413

Cervus_l 4.211097 1.191713 3.534

Equus_caballus_l 1.855188 1.727266 1.074

Myodes_glareolus_l -5.189639 2.070312 -2.507

Lepus_timidus_l -0.820699 2.003581 -0.410

Talpa_europaea_l -3.625931 2.488344 -1.457

Neomys_fodiens_l -2.089003 2.214447 -0.943

Ursus_arctos_l 2.538432 2.096012 1.211


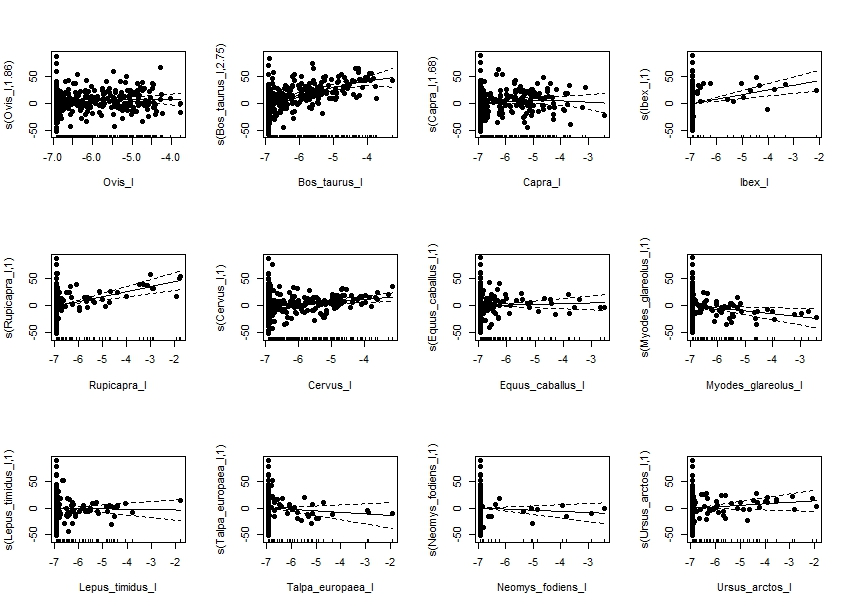


summary(gam.forbs.m1$gam)

Family: gaussian

Link function: identity

Formula:

Forbs_F_richness ~ lake_T + cent_T + lake_P + cent_P + s(Ovis_l) +

s(Bos_taurus_l) + s(Capra_l) + s(Ibex_l) + s(Rupicapra_l) +

s(Cervus_l) + s(Equus_caballus_l) + s(Myodes_glareolus_l) +

s(Lepus_timidus_l) + s(Talpa_europaea_l) + s(Neomys_fodiens_l) +

s(Ursus_arctos_l)

Parametric coefficients:

Estimate Std. Error t value Pr(>|t|)

(Intercept) 88.844561 30.045500 2.957 0.00323 **

lake_T 2.473452 1.660419 1.490 0.13683

cent_T -2.058336 1.109492 -1.855 0.06404 .

lake_P -0.044457 0.019618 -2.266 0.02379 *

cent_P 0.004450 0.008595 0.518 0.60480

---

Signif. codes: 0 ‘***’ 0.001 ‘**’ 0.01 ‘*’ 0.05 ‘.’ 0.1 ‘ ’ 1

Approximate significance of smooth terms:

edf Ref.df F p-value

s(Ovis_l) 1.861 1.861 5.561 0.00295 **

s(Bos_taurus_l) 2.750 2.750 59.562 < 2e-16 ***

s(Capra_l) 1.683 1.683 1.307 0.16310

s(Ibex_l) 1.000 1.000 19.525 1.22e-05 ***

s(Rupicapra_l) 1.000 1.000 29.505 < 2e-16 ***

s(Cervus_l) 1.000 1.000 15.473 9.34e-05 ***

s(Equus_caballus_l) 1.000 1.000 0.635 0.42572

s(Myodes_glareolus_l) 1.000 1.000 7.062 0.00808 **

s(Lepus_timidus_l) 1.000 1.000 0.146 0.70297

s(Talpa_europaea_l) 1.000 1.000 1.346 0.24648

s(Neomys_fodiens_l) 1.000 1.000 0.999 0.31784

s(Ursus_arctos_l) 1.000 1.000 1.863 0.17280

---

Signif. codes: 0 ‘***’ 0.001 ‘**’ 0.01 ‘*’ 0.05 ‘.’ 0.1 ‘ ’ 1

R-sq.(adj) = 0.496

Scale est. = 298.74 n = 637

**Graminoids**

gam.gram.m1 <- gamm(Graminoid_F_richness ~ lake_T + cent_T + lake_P + cent_P + s(Ovis_l) + s(Bos_taurus_l) + s(Capra_l) + s(Ibex_l) + s(Rupicapra_l) + s(Cervus_l)+ s(Equus_caballus_l) + s(Myodes_glareolus_l) + s(Lepus_timidus_l) + s(Talpa_europaea_l) + s(Neomys_fodiens_l) + s(Ursus_arctos_l), random=list(lake_name=~1), data = plantas_RAI_na2)

plot(gam.gram.m1$gam, residuals=T, pages=1, pch=19)

gam.gram.lmer <- lmer(Graminoid_F_richness ~ lake_T + cent_T + lake_P + cent_P + Ovis_l + Bos_taurus_l + Capra_l + Ibex_l + Rupicapra_l + Cervus_l + Equus_caballus_l + Myodes_glareolus_l + Lepus_timidus_l + Talpa_europaea_l + Neomys_fodiens_l + Ursus_arctos_l + (1|lake_name), data = plantas_RAI_na2)

summary(gam.gram.lmer)

Linear mixed model fit by REML ['lmerMod']

Formula: Graminoid_F_richness ~ lake_T + cent_T + lake_P + cent_P + Ovis_l +

Bos_taurus_l + Capra_l + Ibex_l + Rupicapra_l + Cervus_l +

Equus_caballus_l + Myodes_glareolus_l + Lepus_timidus_l +

Talpa_europaea_l + Neomys_fodiens_l + Ursus_arctos_l + (1 | lake_name)

Data: plantas_RAI_na2

REML criterion at convergence: 3316.6

Scaled residuals:

Min 1Q Median 3Q Max

-2.8952 -0.6396 -0.1298 0.6050 3.8986

Random effects:

Groups Name Variance Std.Dev.

lake_name (Intercept) 2.771 1.665

Residual 9.899 3.146

Number of obs: 637, groups: lake_name, 14

Fixed effects:

Estimate Std. Error t value

(Intercept) 55.796089 7.988599 6.984

lake_T -0.271802 0.286991 -0.947

cent_T -1.152008 0.198064 -5.816

lake_P -0.002544 0.003391 -0.750

cent_P 0.001247 0.001554 0.802

Ovis_l 0.458884 0.265689 1.727

Bos_taurus_l 2.986661 0.257822 11.584

Capra_l 0.331509 0.288495 1.149

Ibex_l 1.465273 0.359090 4.081

Rupicapra_l 1.527273 0.311048 4.910

Cervus_l 0.514841 0.210805 2.442

Equus_caballus_l 0.866215 0.306158 2.829

Myodes_glareolus_l -1.353048 0.367032 -3.686

Lepus_timidus_l 0.123807 0.355071 0.349

Talpa_europaea_l -0.538116 0.441314 -1.219

Neomys_fodiens_l -0.358335 0.392710 -0.912

Ursus_arctos_l 0.392424 0.371165 1.057


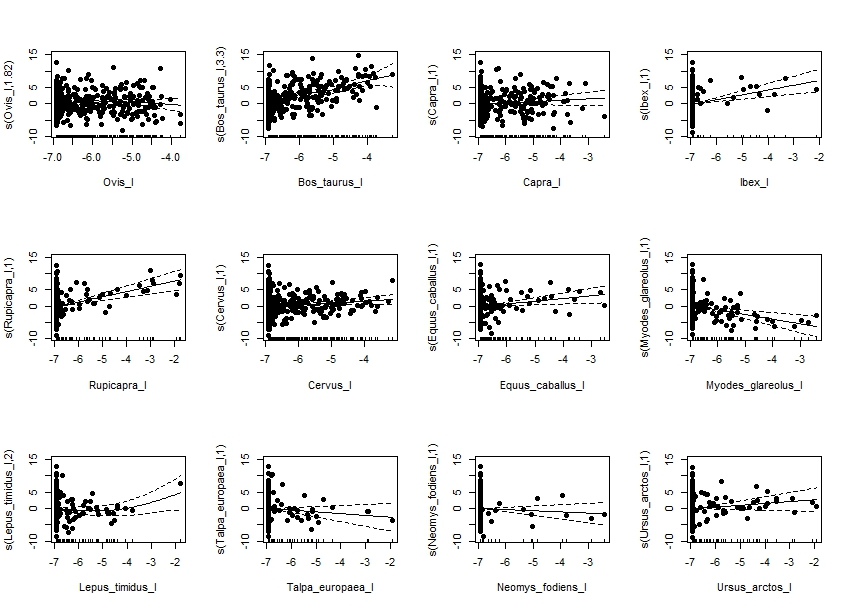


summary(gam.gram.m1$gam)

Family: gaussian

Link function: identity

Formula:

Graminoid_F_richness ~ lake_T + cent_T + lake_P + cent_P + s(Ovis_l) +

s(Bos_taurus_l) + s(Capra_l) + s(Ibex_l) + s(Rupicapra_l) +

s(Cervus_l) + s(Equus_caballus_l) + s(Myodes_glareolus_l) +

s(Lepus_timidus_l) + s(Talpa_europaea_l) + s(Neomys_fodiens_l) +

s(Ursus_arctos_l)

Parametric coefficients:

Estimate Std. Error t value Pr(>|t|)

(Intercept) 13.339279 4.476531 2.980 0.003 **

lake_T -0.284613 0.246301 -1.156 0.248

cent_T -1.013619 0.197077 -5.143 3.63e-07 ***

lake_P -0.002600 0.002922 -0.890 0.374

cent_P 0.001016 0.001522 0.668 0.505

---

Signif. codes: 0 ‘***’ 0.001 ‘**’ 0.01 ‘*’ 0.05 ‘.’ 0.1 ‘ ’ 1

Approximate significance of smooth terms:

edf Ref.df F p-value

s(Ovis_l) 1.821 1.821 1.559 0.13633

s(Bos_taurus_l) 3.299 3.299 43.419 < 2e-16 ***

s(Capra_l) 1.000 1.000 2.243 0.13475

s(Ibex_l) 1.000 1.000 17.657 3.08e-05 ***

s(Rupicapra_l) 1.000 1.000 27.270 4.66e-07 ***

s(Cervus_l) 1.000 1.000 8.365 0.00396 **

s(Equus_caballus_l) 1.000 1.000 7.817 0.00534 **

s(Myodes_glareolus_l) 1.000 1.000 15.465 9.38e-05 ***

s(Lepus_timidus_l) 2.004 2.004 2.173 0.11038

s(Talpa_europaea_l) 1.000 1.000 1.562 0.21191

s(Neomys_fodiens_l) 1.000 1.000 0.849 0.35706

s(Ursus_arctos_l) 1.000 1.000 2.285 0.13112

---

Signif. codes: 0 ‘***’ 0.001 ‘**’ 0.01 ‘*’ 0.05 ‘.’ 0.1 ‘ ’ 1

R-sq.(adj) = 0.386

Scale est. = 9.3879 n = 637

**Abundances within growth forms (RAI)**

**Trees**

gam.trees.RAI.m1 <- gamm(Trees.4 ~ s(lake_T) + s(cent_T) + s(lake_P) + s(cent_P) + s(Ovis_l) + s(Bos_taurus_l) + s(Capra_l) + s(Ibex_l) + s(Rupicapra_l) + s(Cervus_l)+ s(Equus_caballus_l) + s(Myodes_glareolus_l) + s(Lepus_timidus_l) + s(Talpa_europaea_l) + s(Neomys_fodiens_l) + s(Ursus_arctos_l), andom=list(lake_name=~1), data = plantas_RAI_na2)

plot(gam.trees.RAI.m1$gam, residuals=T, pages=1, pch=19)

gam.trees.RAI.lmer <- lmer(Trees.4 ~ lake_T + cent_T + lake_P + cent_P + Ovis_l + Bos_taurus_l + Capra_l + Ibex_l + Rupicapra_l + Cervus_l + Equus_caballus_l + Myodes_glareolus_l + Lepus_timidus_l + Talpa_europaea_l + Neomys_fodiens_l + Ursus_arctos_l + (1|lake_name), data = plantas_RAI_na2)

summary(gam.trees.RAI.lmer)

Linear mixed model fit by REML ['lmerMod']

Formula: Trees.4 ~ lake_T + cent_T + lake_P + cent_P + Ovis_l + Bos_taurus_l +

Capra_l + Ibex_l + Rupicapra_l + Cervus_l + Equus_caballus_l +

Myodes_glareolus_l + Lepus_timidus_l + Talpa_europaea_l + Neomys_fodiens_l + Ursus_arctos_l + (1 | lake_name)

Data: plantas_RAI_na2

REML criterion at convergence: 5042.8

Scaled residuals:

Min 1Q Median 3Q Max

-1.5708 -0.4754 -0.2075 0.0929 6.6993

Random effects:

Groups Name Variance Std.Dev.

lake_name (Intercept) 13.0 3.606

Residual 163.3 12.777

Number of obs: 637, groups: lake_name, 14

Fixed effects:

Estimate Std. Error t value

(Intercept) -96.965694 27.315982 -3.550

lake_T 1.983211 0.683174 2.903

cent_T -1.230738 0.800657 -1.537

lake_P 0.010312 0.008202 1.257

cent_P 0.022133 0.006302 3.512

Ovis_l -2.066620 1.066931 -1.937

Bos_taurus_l -3.186708 1.034075 -3.082

Capra_l -1.623327 1.162869 -1.396

Ibex_l -0.901574 1.453983 -0.620

Rupicapra_l -3.138821 1.251891 -2.507

Cervus_l -1.683198 0.843849 -1.995

Equus_caballus_l 0.182437 1.238905 0.147

Myodes_glareolus_l -0.050795 1.486851 -0.034

Lepus_timidus_l 0.782105 1.435624 0.545

Talpa_europaea_l 0.521189 1.791514 0.291

Neomys_fodiens_l 1.620409 1.593552 1.017

Ursus_arctos_l -0.955381 1.494368 -0.639


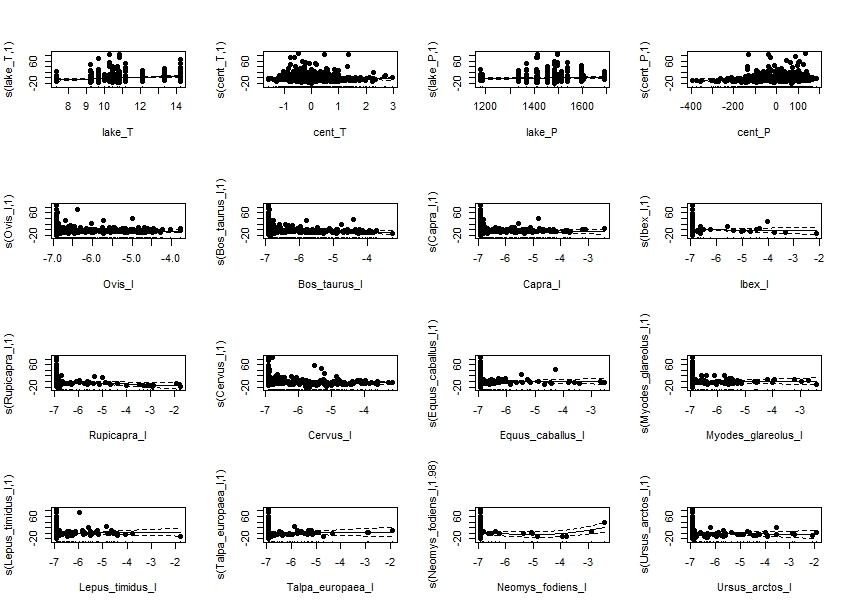


summary(gam.trees.RAI.m1$gam)

Family: gaussian

Link function: identity

Formula:

Trees.4 ~ s(lake_T) + s(cent_T) + s(lake_P) + s(cent_P) + s(Ovis_l) +

s(Bos_taurus_l) + s(Capra_l) + s(Ibex_l) + s(Rupicapra_l) +

s(Cervus_l) + s(Equus_caballus_l) + s(Myodes_glareolus_l) +

s(Lepus_timidus_l) + s(Talpa_europaea_l) + s(Neomys_fodiens_l) +

s(Ursus_arctos_l)

Parametric coefficients:

Estimate Std. Error t value Pr(>|t|)

(Intercept) 9.0018 0.9524 9.452 <2e-16 ***

---

Signif. codes: 0 ‘***’ 0.001 ‘**’ 0.01 ‘*’ 0.05 ‘.’ 0.1 ‘ ’ 1

Approximate significance of smooth terms:

edf Ref.df F p-value

s(lake_T) 1.000 1.000 11.797 0.000633 ***

s(cent_T) 1.000 1.000 2.414 0.120747

s(lake_P) 1.000 1.000 2.051 0.152648

s(cent_P) 1.000 1.000 13.206 0.000303 ***

s(Ovis_l) 1.000 1.000 4.222 0.040330 *

s(Bos_taurus_l) 1.000 1.000 9.095 0.002668 **

s(Capra_l) 1.000 1.000 2.111 0.146750

s(Ibex_l) 1.000 1.000 0.316 0.574159

s(Rupicapra_l) 1.000 1.000 6.474 0.011191 *

s(Cervus_l) 1.000 1.000 4.059 0.044358 *

s(Equus_caballus_l) 1.000 1.000 0.008 0.927387

s(Myodes_glareolus_l) 1.000 1.000 0.001 0.975972

s(Lepus_timidus_l) 1.000 1.000 0.292 0.589262

s(Talpa_europaea_l) 1.000 1.000 0.212 0.645213

s(Neomys_fodiens_l) 1.978 1.978 2.600 0.091860 .

s(Ursus_arctos_l) 1.000 1.000 0.465 0.495479

---

Signif. codes: 0 ‘***’ 0.001 ‘**’ 0.01 ‘*’ 0.05 ‘.’ 0.1 ‘ ’ 1

R-sq.(adj) = 0.152

Scale est. = 158.59 n = 637

**Shrubs**

gam.shrubs.RAI.m1 <- gamm(Shrubs.4 ~ s(lake_T) + s(cent_T) + s(lake_P) + s(cent_P) + s(Ovis_l) + s(Bos_taurus_l) + s(Capra_l) + s(Ibex_l) + s(Rupicapra_l) + s(Cervus_l) + s(Equus_caballus_l) + s(Myodes_glareolus_l) + s(Lepus_timidus_l) + s(Talpa_europaea_l) + s(Neomys_fodiens_l) + s(Ursus_arctos_l), random=list(lake_name=~1), data = plantas_RAI_na2)

plot(gam.shrubs.RAI.m1$gam, residuals=T, pages=1, pch=19)

gam.shrubs.lmer <- lmer(Shrubs.4 ~ lake_T + cent_T + lake_P + cent_P + Ovis_l + Bos_taurus_l + Capra_l + Ibex_l + Rupicapra_l + Cervus_l + Equus_caballus_l + Myodes_glareolus_l + Lepus_timidus_l + Talpa_europaea_l + Neomys_fodiens_l + Ursus_arctos_l + (1|lake_name), data = plantas_RAI_na2)

summary(gam.shrubs.lmer)

Linear mixed model fit by REML ['lmerMod']

Formula: Shrubs.4 ~ lake_T + cent_T + lake_P + cent_P + Ovis_l + Bos_taurus_l +

Capra_l + Ibex_l + Rupicapra_l + Cervus_l + Equus_caballus_l +

Myodes_glareolus_l + Lepus_timidus_l + Talpa_europaea_l + Neomys_fodiens_l + Ursus_arctos_l + (1 | lake_name)

Data: plantas_RAI_na2

REML criterion at convergence: 5122

Scaled residuals:

Min 1Q Median 3Q Max

-2.2037 -0.5431 -0.2115 0.2351 6.0408

Random effects:

Groups Name Variance Std.Dev.

lake_name (Intercept) 70.77 8.412

Residual 181.09 13.457

Number of obs: 637, groups: lake_name, 14

Fixed effects:

Estimate Std. Error t value

(Intercept) -1.055e+02 3.669e+01 -2.874

lake_T 1.235e+00 1.433e+00 0.862

cent_T 2.250e+00 8.477e-01 2.654

lake_P 2.410e-02 1.690e-02 1.426

cent_P 2.074e-02 6.648e-03 3.119

Ovis_l 2.803e+00 1.138e+00 2.463

Bos_taurus_l -3.348e+00 1.105e+00 -3.031

Capra_l 6.910e-01 1.235e+00 0.559

Ibex_l -4.238e+00 1.537e+00 -2.758

Rupicapra_l -2.778e+00 1.332e+00 -2.085

Cervus_l -9.410e-01 9.035e-01 -1.042

Equus_caballus_l -2.206e+00 1.310e+00 -1.684

Myodes_glareolus_l 7.707e-01 1.570e+00 0.491

Lepus_timidus_l 5.420e-02 1.520e+00 0.036

Talpa_europaea_l 4.056e-01 1.888e+00 0.215

Neomys_fodiens_l 3.779e-01 1.680e+00 0.225

Ursus_arctos_l -2.016e+00 1.590e+00 -1.268


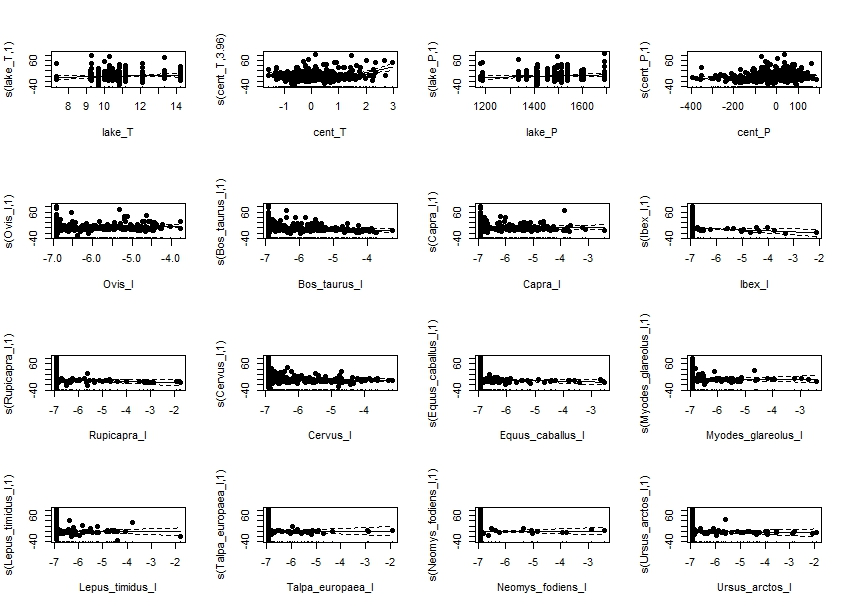


summary(gam.shrubs.RAI.m1$gam)

Family: gaussian

Link function: identity

Formula:

Shrubs.4 ~ s(lake_T) + s(cent_T) + s(lake_P) + s(cent_P) + s(Ovis_l) +

s(Bos_taurus_l) + s(Capra_l) + s(Ibex_l) + s(Rupicapra_l) +

s(Cervus_l) + s(Equus_caballus_l) + s(Myodes_glareolus_l) +

s(Lepus_timidus_l) + s(Talpa_europaea_l) + s(Neomys_fodiens_l) +

s(Ursus_arctos_l)

Parametric coefficients:

Estimate Std. Error t value Pr(>|t|)

(Intercept) 14.255 2.018 7.065 4.35e-12 ***

---

Signif. codes: 0 ‘***’ 0.001 ‘**’ 0.01 ‘*’ 0.05 ‘.’ 0.1 ‘ ’ 1

Approximate significance of smooth terms:

edf Ref.df F p-value

s(lake_T) 1.000 1.000 1.212 0.271335

s(cent_T) 3.959 3.959 9.200 1.08e-06 ***

s(lake_P) 1.000 1.000 1.979 0.160025

s(cent_P) 1.000 1.000 11.508 0.000737 ***

s(Ovis_l) 1.000 1.000 5.585 0.018427 *

s(Bos_taurus_l) 1.000 1.000 7.780 0.005445 **

s(Capra_l) 1.000 1.000 0.148 0.700375

s(Ibex_l) 1.000 1.000 7.148 0.007703 **

s(Rupicapra_l) 1.000 1.000 3.125 0.077614 .

s(Cervus_l) 1.000 1.000 0.949 0.330413

s(Equus_caballus_l) 1.000 1.000 3.218 0.073317 .

s(Myodes_glareolus_l) 1.000 1.000 0.128 0.720379

s(Lepus_timidus_l) 1.000 1.000 0.041 0.840568

s(Talpa_europaea_l) 1.000 1.000 0.006 0.936654

s(Neomys_fodiens_l) 1.000 1.000 0.013 0.907595

s(Ursus_arctos_l) 1.000 1.000 0.572 0.449819

---

Signif. codes: 0 ‘***’ 0.001 ‘**’ 0.01 ‘*’ 0.05 ‘.’ 0.1 ‘ ’ 1

R-sq.(adj) = 0.14

Scale est. = 169.17 n = 637

**Forbs**

gam.forbs.RAI.m1 <- gamm(Forbs.4 ~ s(lake_T) + s(cent_T) + s(lake_P) + s(cent_P) + s(Ovis_l) + s(Bos_taurus_l) + s(Capra_l) + s(Ibex_l) + s(Rupicapra_l) + s(Cervus_l) + s(Equus_caballus_l) + s(Myodes_glareolus_l) + s(Lepus_timidus_l) + s(Talpa_europaea_l) + s(Neomys_fodiens_l) + s(Ursus_arctos_l), random=list(lake_name=~1), data = plantas_RAI_na2)

plot(gam.forbs.RAI.m1$gam, residuals=T, pages=1, pch=19)

gam.forbs.lmer <- lmer(Forbs.4 ~ lake_T + cent_T + lake_P + cent_P + Ovis_l + Bos_taurus_l + Capra_l + Ibex_l + Rupicapra_l + Cervus_l + Equus_caballus_l + Myodes_glareolus_l + Lepus_timidus_l + Talpa_europaea_l + Neomys_fodiens_l + Ursus_arctos_l + (1|lake_name), data = plantas_RAI_na2)

ummary(gam.forbs.lmer)

Linear mixed model fit by REML ['lmerMod']

Formula: Forbs.4 ~ lake_T + cent_T + lake_P + cent_P + Ovis_l + Bos_taurus_l +

Capra_l + Ibex_l + Rupicapra_l + Cervus_l + Equus_caballus_l +

Myodes_glareolus_l + Lepus_timidus_l + Talpa_europaea_l + Neomys_fodiens_l + Ursus_arctos_l + (1 | lake_name)

Data: plantas_RAI_na2

REML criterion at convergence: 5548.2

Scaled residuals:

Min 1Q Median 3Q Max

-3.9116 -0.4277 0.1956 0.6537 2.4979

Random effects:

Groups Name Variance Std.Dev.

lake_name (Intercept) 109.5 10.46

Residual 361.6 19.02

Number of obs: 637, groups: lake_name, 14

Fixed effects:

Estimate Std. Error t value

(Intercept) 252.684027 49.044733 5.152

lake_T -1.595108 1.798217 -0.887

cent_T 0.347471 1.197344 0.290

lake_P -0.028320 0.021238 -1.333

cent_P -0.041886 0.009393 -4.459

Ovis_l 0.229476 1.606533 0.143

Bos_taurus_l 6.383676 1.559053 4.095

Capra_l 0.923138 1.744200 0.529

Ibex_l 4.703065 2.170646 2.167

Rupicapra_l 5.745714 1.880670 3.055

Cervus_l 1.925157 1.274834 1.510

Equus_caballus_l 0.887726 1.850722 0.480

Myodes_glareolus_l 0.602680 2.218621 0.272

Lepus_timidus_l -3.361604 2.146476 -1.566

Talpa_europaea_l -1.469651 2.667427 -0.551

Neomys_fodiens_l -1.589106 2.373683 -0.669

Ursus_arctos_l 3.414569 2.244118 1.522


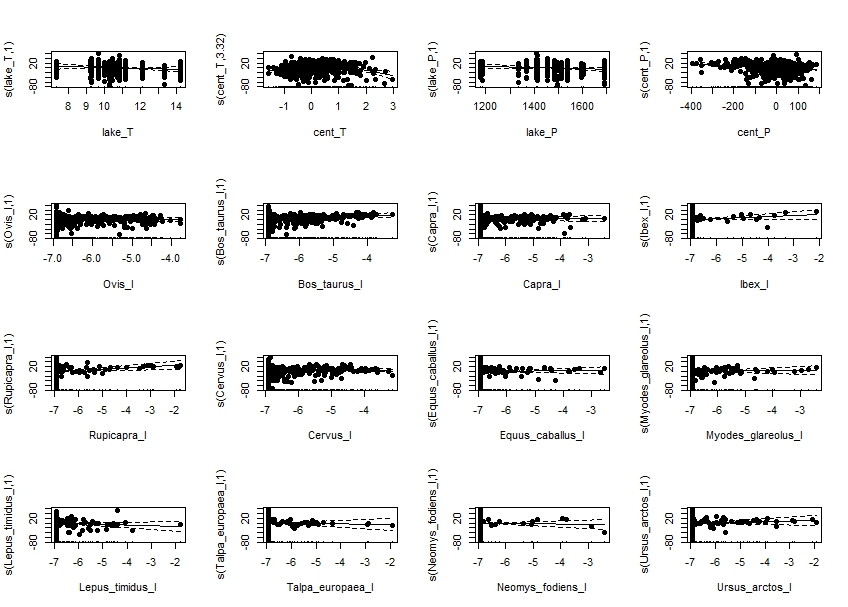


summary(gam.forbs.RAI.m1$gam)

Family: gaussian

Link function: identity

Formula:

Forbs.4 ~ s(lake_T) + s(cent_T) + s(lake_P) + s(cent_P) + s(Ovis_l) +

s(Bos_taurus_l) + s(Capra_l) + s(Ibex_l) + s(Rupicapra_l) +

s(Cervus_l) + s(Equus_caballus_l) + s(Myodes_glareolus_l) +

s(Lepus_timidus_l) + s(Talpa_europaea_l) + s(Neomys_fodiens_l) +

s(Ursus_arctos_l)

Parametric coefficients:

Estimate Std. Error t value Pr(>|t|)

(Intercept) 71.016 2.625 27.06 <2e-16 ***

---

Signif. codes: 0 ‘***’ 0.001 ‘**’ 0.01 ‘*’ 0.05 ‘.’ 0.1 ‘ ’ 1

Approximate significance of smooth terms:

edf Ref.df F p-value

s(lake_T) 1.000 1.000 1.306 0.253640

s(cent_T) 3.318 3.318 7.039 9.23e-05 ***

s(lake_P) 1.000 1.000 1.576 0.209875

s(cent_P) 1.000 1.000 23.515 1.75e-06 ***

s(Ovis_l) 1.000 1.000 0.058 0.810008

s(Bos_taurus_l) 1.000 1.000 15.323 0.000101 ***

s(Capra_l) 1.000 1.000 0.580 0.446439

s(Ibex_l) 1.000 1.000 4.507 0.034148 *

s(Rupicapra_l) 1.000 1.000 8.425 0.003832 **

s(Cervus_l) 1.000 1.000 2.064 0.151317

s(Equus_caballus_l) 1.000 1.000 0.279 0.597457

s(Myodes_glareolus_l) 1.000 1.000 0.262 0.609127

s(Lepus_timidus_l) 1.000 1.000 1.917 0.166652

s(Talpa_europaea_l) 1.000 1.000 0.188 0.664634

s(Neomys_fodiens_l) 1.000 1.000 0.327 0.567828

s(Ursus_arctos_l) 1.000 1.000 1.130 0.288138

---

Signif. codes: 0 ‘***’ 0.001 ‘**’ 0.01 ‘*’ 0.05 ‘.’ 0.1 ‘ ’ 1

R-sq.(adj) = 0.169

Scale est. = 340.54 n = 637

**Graminoids**

gam.graminoids.RAI.m1 <- gamm(Graminoid.4 ~ s(lake_T) + s(cent_T) + s(lake_P) + s(cent_P) + s(Ovis_l) + s(Bos_taurus_l) + s(Capra_l) + s(Ibex_l) + s(Rupicapra_l) + s(Cervus_l)+ s(Equus_caballus_l) + s(Myodes_glareolus_l) + s(Lepus_timidus_l) + s(Talpa_europaea_l) + s(Neomys_fodiens_l) + s(Ursus_arctos_l), random=list(lake_name=~1), data = plantas_RAI_na2)

plot(gam.graminoids.RAI.m1$gam, residuals=T, pages=1, pch=19)

gam.graminoids.lmer <- lmer(Graminoid.4 ~ lake_T + cent_T + lake_P + cent_P + Ovis_l + Bos_taurus_l + Capra_l + Ibex_l + Rupicapra_l + Cervus_l + Equus_caballus_l + Myodes_glareolus_l + Lepus_timidus_l + Talpa_europaea_l + Neomys_fodiens_l + Ursus_arctos_l + (1|lake_name), data = plantas_RAI_na2)

summary(gam.graminoids.lmer)

Linear mixed model fit by REML ['lmerMod']

Formula: Graminoid.4 ~ lake_T + cent_T + lake_P + cent_P + Ovis_l + Bos_taurus_l +

Capra_l + Ibex_l + Rupicapra_l + Cervus_l + Equus_caballus_l +

Myodes_glareolus_l + Lepus_timidus_l + Talpa_europaea_l + Neomys_fodiens_l + Ursus_arctos_l + (1 | lake_name)

Data: plantas_RAI_na2

REML criterion at convergence: 4613

Scaled residuals:

Min 1Q Median 3Q Max

-1.4996 -0.4436 -0.1850 0.0975 8.2120

Random effects:

Groups Name Variance Std.Dev.

lake_name (Intercept) 7.229 2.689

Residual 81.500 9.028

Number of obs: 637, groups: lake_name, 14

Fixed effects:

Estimate Std. Error t value

(Intercept) 45.7360400 19.4965869 2.346

lake_T -1.5660652 0.5032077 -3.112

cent_T -1.4287800 0.5660116 -2.524

lake_P -0.0063295 0.0060291 -1.050

cent_P -0.0004203 0.0044535 -0.094

Ovis_l -1.1918846 0.7548710 -1.579

Bos_taurus_l 0.2189007 0.7317029 0.299

Capra_l 0.0225895 0.8223614 0.027

Ibex_l 0.5882113 1.0276805 0.572

Rupicapra_l 0.1423516 0.8854634 0.161

Cervus_l 0.5543163 0.5972393 0.928

Equus_caballus_l 1.0284795 0.8757244 1.174

Myodes_glareolus_l -1.4003811 1.0508435 -1.333

Lepus_timidus_l 2.4935131 1.0148642 2.457

Talpa_europaea_l 0.4417632 1.2658529 0.349

Neomys_fodiens_l -0.4684789 1.1260340 -0.416

Ursus_arctos_l -0.4605809 1.0569243 -0.436


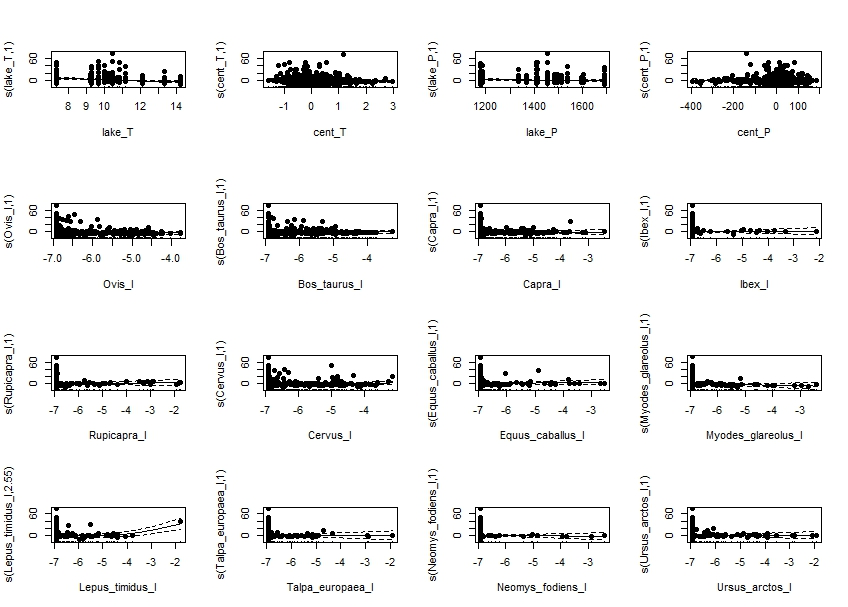


summary(gam.graminoids.RAI.m1$gam)

Family: gaussian

Link function: identity

Formula:

Graminoid.4 ~ s(lake_T) + s(cent_T) + s(lake_P) + s(cent_P) +

s(Ovis_l) + s(Bos_taurus_l) + s(Capra_l) + s(Ibex_l) + s(Rupicapra_l) +

s(Cervus_l) + s(Equus_caballus_l) + s(Myodes_glareolus_l) +

s(Lepus_timidus_l) + s(Talpa_europaea_l) + s(Neomys_fodiens_l) +

s(Ursus_arctos_l)

Parametric coefficients:

Estimate Std. Error t value Pr(>|t|)

(Intercept) 5.7087 0.6975 8.185 1.56e-15 ***

---

Signif. codes: 0 ‘***’ 0.001 ‘**’ 0.01 ‘*’ 0.05 ‘.’ 0.1 ‘ ’ 1

Approximate significance of smooth terms:

edf Ref.df F p-value

s(lake_T) 1.000 1.000 12.903 0.000355 ***

s(cent_T) 1.000 1.000 5.762 0.016670 *

s(lake_P) 1.000 1.000 1.504 0.220477

s(cent_P) 1.000 1.000 0.010 0.921566

s(Ovis_l) 1.000 1.000 1.884 0.170416

s(Bos_taurus_l) 1.000 1.000 0.082 0.774859

s(Capra_l) 1.000 1.000 0.004 0.947842

s(Ibex_l) 1.000 1.000 0.248 0.618398

s(Rupicapra_l) 1.000 1.000 0.434 0.510080

s(Cervus_l) 1.000 1.000 1.029 0.310812

s(Equus_caballus_l) 1.000 1.000 1.555 0.212873

s(Myodes_glareolus_l) 1.000 1.000 2.090 0.148756

s(Lepus_timidus_l) 2.546 2.546 4.314 0.004536 **

s(Talpa_europaea_l) 1.000 1.000 0.004 0.950331

s(Neomys_fodiens_l) 1.000 1.000 0.205 0.651201

s(Ursus_arctos_l) 1.000 1.000 0.010 0.918649

---

Signif. codes: 0 ‘***’ 0.001 ‘**’ 0.01 ‘*’ 0.05 ‘.’ 0.1 ‘ ’ 1

R-sq.(adj) = 0.114

Scale est. = 78.644 n = 637

**Light indicator taxa**

gam.light.m1 <- gamm(Light ~ lake_T + cent_T + lake_P + cent_P + s(Ovis_l) + s(Bos_taurus_l) + s(Capra_l)+ s(Ibex_l) + s(Rupicapra_l) + s(Cervus_l)+ s(Equus_caballus_l) + s(Myodes_glareolus_l) + s(Lepus_timidus_l) + s(Talpa_europaea_l) + s(Neomys_fodiens_l) + s(Ursus_arctos_l), random=list(lake_name=~1), data = plantas_RAI_na2)

plot(gam.light.m1$gam, residuals=T, pages=1, pch=19)

gam.light.lmer <- lmer(Light ~ lake_T + cent_T + lake_P + cent_P + Ovis_l + Bos_taurus_l + Capra_l + Ibex_l + Rupicapra_l + Cervus_l + Equus_caballus_l + Myodes_glareolus_l + Lepus_timidus_l + Talpa_europaea_l + Neomys_fodiens_l + Ursus_arctos_l + (1|lake_name), data = plantas_RAI_na2)

summary(gam.light.lmer)

Linear mixed model fit by REML ['lmerMod']

Formula: Light ~ lake_T + cent_T + lake_P + cent_P + Ovis_l + Bos_taurus_l +

Capra_l + Ibex_l + Rupicapra_l + Cervus_l + Equus_caballus_l +

Myodes_glareolus_l + Lepus_timidus_l + Talpa_europaea_l + Neomys_fodiens_l + Ursus_arctos_l + (1 | lake_name)

Data: plantas_RAI_na2

REML criterion at convergence: 5735.8

Scaled residuals:

Min 1Q Median 3Q Max

-3.1618 -0.6004 -0.0123 0.6815 3.9943

Random effects:

Groups Name Variance Std.Dev.

lake_name (Intercept) 451.1 21.24

Residual 480.1 21.91

Number of obs: 637, groups: lake_name, 14

Fixed effects:

Estimate Std. Error t value

(Intercept) 353.26681 76.74892 4.603

lake_T -3.19520 3.55050 -0.900

cent_T 0.08838 1.38182 0.064

lake_P -0.10093 0.04174 -2.418

cent_P -0.07540 0.01083 -6.963

Ovis_l -2.50704 1.85786 -1.349

Bos_taurus_l 8.73976 1.80398 4.845

Capra_l -0.92292 2.01476 -0.458

Ibex_l 8.20732 2.50359 3.278

Rupicapra_l 9.71611 2.17359 4.470

Cervus_l -2.06614 1.47606 -1.400

Equus_caballus_l 4.28309 2.13505 2.006

Myodes_glareolus_l -1.99471 2.55860 -0.780

Lepus_timidus_l -1.93283 2.47706 -0.780

Talpa_europaea_l -3.21265 3.07403 -1.045

Neomys_fodiens_l -2.83947 2.73585 -1.038

Ursus_arctos_l 2.50378 2.59334 0.965


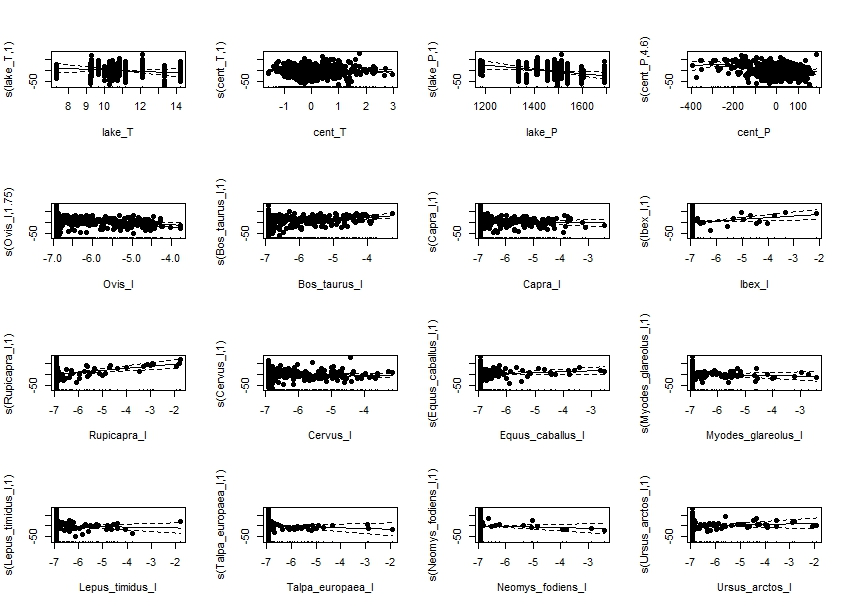


summary(gam.light.m1$gam)

Family: gaussian

Link function: identity

Formula:

Light ~ s(lake_T) + s(cent_T) + s(lake_P) + s(cent_P) + s(Ovis_l) +

s(Bos_taurus_l) + s(Capra_l) + s(Ibex_l) + s(Rupicapra_l) +

s(Cervus_l) + s(Equus_caballus_l) + s(Myodes_glareolus_l) +

s(Lepus_timidus_l) + s(Talpa_europaea_l) + s(Neomys_fodiens_l) +

s(Ursus_arctos_l)

Parametric coefficients:

Estimate Std. Error t value Pr(>|t|)

(Intercept) 49.869 4.916 10.14 <2e-16 ***

---

Signif. codes: 0 ‘***’ 0.001 ‘**’ 0.01 ‘*’ 0.05 ‘.’ 0.1 ‘ ’ 1

Approximate significance of smooth terms:

edf Ref.df F p-value

s(lake_T) 0.9999 0.9999 1.183 0.27718

s(cent_T) 1.0000 1.0000 0.437 0.50863

s(lake_P) 1.0001 1.0001 8.050 0.00470 **

s(cent_P) 4.5962 4.5962 14.137 < 2e-16 ***

s(Ovis_l) 1.7477 1.7477 2.910 0.14842

s(Bos_taurus_l) 1.0000 1.0000 25.706 9.12e-07 ***

s(Capra_l) 1.0000 1.0000 0.066 0.79720

s(Ibex_l) 1.0000 1.0000 9.400 0.00226 **

s(Rupicapra_l) 1.0000 1.0000 20.636 6.88e-06 ***

s(Cervus_l) 1.0000 1.0000 1.620 0.20362

s(Equus_caballus_l) 1.0000 1.0000 3.534 0.06058 .

s(Myodes_glareolus_l) 1.0000 1.0000 0.871 0.35092

s(Lepus_timidus_l) 1.0000 1.0000 0.646 0.42201

s(Talpa_europaea_l) 1.0000 1.0000 1.113 0.29184

s(Neomys_fodiens_l) 1.0000 1.0000 1.120 0.29025

s(Ursus_arctos_l) 1.0000 1.0000 0.897 0.34390

---

Signif. codes: 0 ‘***’ 0.001 ‘**’ 0.01 ‘*’ 0.05 ‘.’ 0.1 ‘ ’ 1

R-sq.(adj) = 0.316

Scale est. = 458.25 n = 637

**Grazing**

gam.grazing.m1 <- gamm(tolerant ~ lake_T + cent_T + lake_P + cent_P + s(Ovis_l) + s(Bos_taurus_l) + s(Capra_l)+ s(Ibex_l) + s(Rupicapra_l) + s(Cervus_l)+ s(Equus_caballus_l) + s(Myodes_glareolus_l) + s(Lepus_timidus_l) + s(Talpa_europaea_l) + s(Neomys_fodiens_l) + s(Ursus_arctos_l), random=list(lake_name=~1), data = plantas_RAI_na2)

plot(gam.grazing.m1$gam, residuals=T, pages=1, pch=19)

gam.grazing.lmer <- lmer(tolerant ~ lake_T + cent_T + lake_P + cent_P + Ovis_l + Bos_taurus_l + Capra_l + Ibex_l + Rupicapra_l + Cervus_l + Equus_caballus_l + Myodes_glareolus_l + Lepus_timidus_l + Talpa_europaea_l + Neomys_fodiens_l + Ursus_arctos_l + (1|lake_name), data = plantas_RAI_na2)

summary(gam.grazing.lmer)

Linear mixed model fit by REML ['lmerMod']

Formula: tolerant ~ lake_T + cent_T + lake_P + cent_P + Ovis_l + Bos_taurus_l +

Capra_l + Ibex_l + Rupicapra_l + Cervus_l + Equus_caballus_l +

Myodes_glareolus_l + Lepus_timidus_l + Talpa_europaea_l + Neomys_fodiens_l + Ursus_arctos_l + (1 | lake_name)

Data: plantas_RAI_na2

REML criterion at convergence: 5698.3

Scaled residuals:

Min 1Q Median 3Q Max

-4.0311 -0.4047 0.1961 0.6061 1.9610

Random effects:

Groups Name Variance Std.Dev.

lake_name (Intercept) 171.2 13.08

Residual 459.1 21.43

Number of obs: 637, groups: lake_name, 14

Fixed effects:

Estimate Std. Error t value

(Intercept) 230.77912 57.79985 3.993

lake_T -2.44006 2.23186 -1.093

cent_T -1.44289 1.34967 -1.069

lake_P -0.04651 0.02633 -1.767

cent_P -0.02273 0.01058 -2.148

Ovis_l -4.56932 1.81191 -2.522

Bos_taurus_l 4.90116 1.75861 2.787

Capra_l -0.10276 1.96657 -0.052

Ibex_l 0.83417 2.44641 0.341

Rupicapra_l 4.09724 2.12074 1.932

Cervus_l 1.68228 1.43826 1.170

Equus_caballus_l 1.87850 2.08596 0.901

Myodes_glareolus_l -0.63079 2.50039 -0.252

Lepus_timidus_l -3.18556 2.41951 -1.317

Talpa_europaea_l 1.58624 3.00564 0.528

Neomys_fodiens_l -0.92569 2.67474 -0.346

Ursus_arctos_l 3.28111 2.53051 1.297


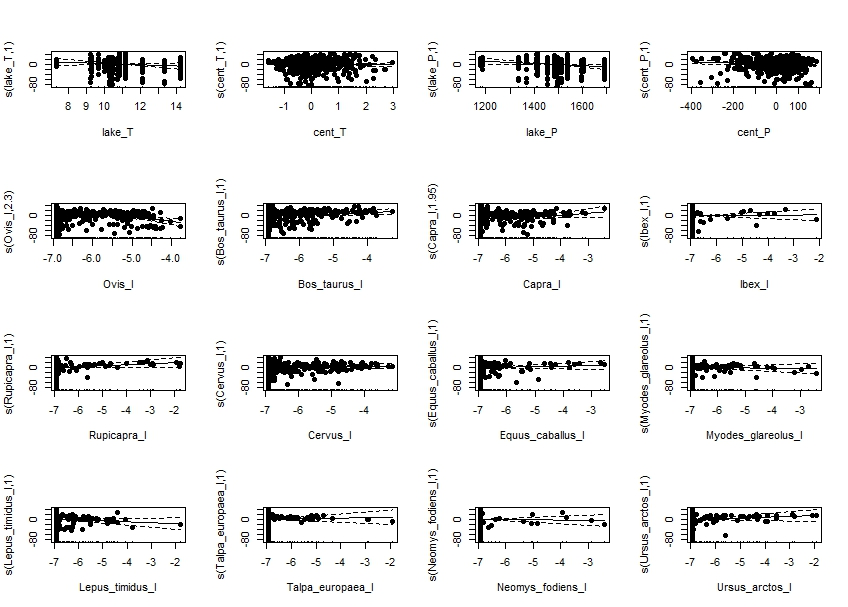


summary(gam.grazing.m1$gam)

Family: gaussian

Link function: identity

Formula:

tolerant ~ s(lake_T) + s(cent_T) + s(lake_P) + s(cent_P) + s(Ovis_l) +

s(Bos_taurus_l) + s(Capra_l) + s(Ibex_l) + s(Rupicapra_l) +

s(Cervus_l) + s(Equus_caballus_l) + s(Myodes_glareolus_l) +

s(Lepus_timidus_l) + s(Talpa_europaea_l) + s(Neomys_fodiens_l) +

s(Ursus_arctos_l)

Parametric coefficients:

Estimate Std. Error t value Pr(>|t|)

(Intercept) 76.316 3.182 23.99 <2e-16 ***

---

Signif. codes: 0 ‘***’ 0.001 ‘**’ 0.01 ‘*’ 0.05 ‘.’ 0.1 ‘ ’ 1

Approximate significance of smooth terms:

edf Ref.df F p-value

s(lake_T) 1.000 1.000 1.563 0.21169

s(cent_T) 1.000 1.000 0.578 0.44756

s(lake_P) 1.000 1.000 3.662 0.05613 .

s(cent_P) 1.000 1.000 4.572 0.03289 *

s(Ovis_l) 2.297 2.297 5.530 0.00233 **

s(Bos_taurus_l) 1.000 1.000 8.209 0.00431 **

s(Capra_l) 1.952 1.953 2.152 0.10476

s(Ibex_l) 1.000 1.000 0.121 0.72800

s(Rupicapra_l) 1.000 1.000 3.847 0.05028 .

s(Cervus_l) 1.000 1.000 1.894 0.16922

s(Equus_caballus_l) 1.000 1.000 1.141 0.28581

s(Myodes_glareolus_l) 1.000 1.000 0.096 0.75675

s(Lepus_timidus_l) 1.000 1.000 2.193 0.13916

s(Talpa_europaea_l) 1.000 1.000 0.334 0.56332

s(Neomys_fodiens_l) 1.000 1.000 0.135 0.71301

s(Ursus_arctos_l) 1.000 1.000 1.598 0.20664

---

Signif. codes: 0 ‘***’ 0.001 ‘**’ 0.01 ‘*’ 0.05 ‘.’ 0.1 ‘ ’ 1

R-sq.(adj) = 0.147

Scale est. = 440.99 n = 637
