# Supplementary material for: Wild and domesticated animal abundance is associated with greater late-Holocene alpine plant diversity
Source: Nat Commun. 2025 Apr 25;16:3924. doi: 10.1038/s41467-025-59028-2 (PMC12032255; doi:10.1038/s41467-025-59028-2)
Supplement: Supplementary file 5 — Reporting Summary [file 41467_2025_59028_MOESM5_ESM.pdf]

nature portfolio

Corresponding author(s): Sandra Garcés-Pastor

Last updated by author(s): Feb 21, 2025

Reporting Summary

Nature Portfolio wishes to improve the reproducibility of the work that we publish. This form provides structure for consistency and transparency in reporting. For further information on Nature Portfolio policies, see our Editorial Policies and the Editorial Policy Checklist.

Statistics

For all statistical analyses, confirm that the following items are present in the figure legend, table legend, main text, or Methods section.

n/a	Confirmed	
☐	☒	The exact sample size (*n*) for each experimental group/condition, given as a discrete number and unit of measurement
☐	☒	A statement on whether measurements were taken from distinct samples or whether the same sample was measured repeatedly
☒	☐	The statistical test(s) used AND whether they are one- or two-sided<br>*Only common tests should be described solely by name; describe more complex techniques in the Methods section.*
☒	☐	A description of all covariates tested
☒	☐	A description of any assumptions or corrections, such as tests of normality and adjustment for multiple comparisons
☒	☐	A full description of the statistical parameters including central tendency (e.g. means) or other basic estimates (e.g. regression coefficient) AND variation (e.g. standard deviation) or associated estimates of uncertainty (e.g. confidence intervals)
☒	☐	For null hypothesis testing, the test statistic (e.g. *F*, *t*, *r*) with confidence intervals, effect sizes, degrees of freedom and *P* value noted<br>*Give P values as exact values whenever suitable.*
☒	☐	For Bayesian analysis, information on the choice of priors and Markov chain Monte Carlo settings
☒	☐	For hierarchical and complex designs, identification of the appropriate level for tests and full reporting of outcomes
☒	☐	Estimates of effect sizes (e.g. Cohen's *d*, Pearson's *r*), indicating how they were calculated

Our web collection on statistics for biologists contains articles on many of the points above.

Software and code

Policy information about availability of computer code

Data collection	Does not apply
Data analysis	OBITools software package was used to analyse the sequence data. The identified sequences were filtered in R using a custom script (available at https://github.com/Y-Lammers/MergeAndFilter). Plots were made with R v4.4.0 using the vegan, rioja and ggplot2 packages 68–70.

For manuscripts utilizing custom algorithms or software that are central to the research but not yet described in published literature, software must be made available to editors and reviewers. We strongly encourage code deposition in a community repository (e.g. GitHub). See the Nature Portfolio guidelines for submitting code & software for further information.

Data

Policy information about availability of data

All manuscripts must include a data availability statement. This statement should provide the following information, where applicable:

- Accession codes, unique identifiers, or web links for publicly available datasets
- A description of any restrictions on data availability
- For clinical datasets or third party data, please ensure that the statement adheres to our policy

The lookup dataset for all tag and sample combinations generated in this study have been deposited at the Zenodo repository (https://doi.org/10.5281/zenodo.14283341). The raw OBITools output data both the P6-loop (all reference libraries) and 16s datasets are available at https://filesender.sikt.no/?s=download&token=1f99961d-43c4-4326-a508-4ee48a5a7f80.
The Source Data for Figures 1, 2, 3 and Supplementary Figures 1 to 11 generated in this study are provided in the Supplementary Data 12.

Field-specific reporting

Please select the one below that is the best fit for your research. If you are not sure, read the appropriate sections before making your selection.

☐ Life sciences ☐ Behavioural & social sciences ☒ Ecological, evolutionary & environmental sciences

For a reference copy of the document with all sections, see nature.com/documents/nr-reporting-summary-flat.pdf

Ecological, evolutionary & environmental sciences study design

All studies must disclose on these points even when the disclosure is negative.

Study description	Here, we reconstruct the richest plant diversity and mammalian distributions across the Alps during the Holocene, employing the largest lake sedaDNA dataset to date. We applied sedaDNA analysis to reveal how climate, animals and humans shaped plant diversity in different areas of the Alps. We also investigated the impact of large herbivores and climate on the diversity and abundance of alpine species, specifically forbs and graminoids, shedding light on the effects of grazing behaviour on plant diversity.
Research sample	14 lake cores were retrieved and opened by longitudinal splitting. One half was used for proxy subsampling, and the other half was used for photography.
Sampling strategy	Cores were subsampled for sedaDNA every 4-12 cm, with the interval dependent on total core length, by taking a 1 cm thick longitudinal subsample while avoiding the first 4–10 mm of exposed sediment 50. Subsampling was performed in the ancient DNA lab at TMU using sterile tools, a full bodysuit, and gloves to ensure uncontaminated samples. For Sulzkar, subsampling was conducted in a cleaned teaching lab where no molecular biology work had been previously carried out at the Department of Ecology, University of Innsbruck in Austria.
Data collection	Inger Greve Alsos, Peter D. Heintzman, Youri Lammers, Sandra Garcés-Pastor, Christoph Schwörer, Lieveke van Vugt, Willy Tinner, Fabian Rey, Boris Vannière, Andreas Tribsch, Charline Giguet-Covex, Robert Schabetsberger and Kevin Walsh did the fieldwork; Sandra Garcés-Pastor and Scarlett Zetter did the ancient DNA laboratory work with input from Inger Greve Alsos and Peter D. Heintzman; Data was collected in a shared online document that was accessible for all the coauthors. Tomasz Goslar performed radiocarbon dating; Christoph Schwörer built composite cores and performed age-depth modeling.
Timing and spatial scale	All DNA samples were sampled and extracted at the same time
Data exclusions	During the analysis 67 samples for plants with low quality were removed, , which had technical quality (MTQ) scores <0.52 and/or analytical quality (MAQ) scores <0.25. All samples, including the ones that were removed, are included in the raw data.
Reproducibility	Raw data will be made available and can be used to reproduce the results. The experiment did not failed and we did not have any attempt to repeat it.
Randomization	Randomization is not applicable to this study
Blinding	Blinding is not applicable to this study

Did the study involve field work? ☒ Yes ☐ No

Field work, collection and transport

Field conditions	Environmental onditions in the field work were irrelevant to the study question or for the sediment preservation. Once it was retrieved it was stored in cold conditions.
Location	The 14 lakes of this study cover almost all the countries that comprise the Alps: four from Austria, three from Italy, four from Switzerland and three from France. The clocations of the 14 lakes are available in the Supplementary Table 1.
Access & import/export	Cores were transported to the Arctic University Museum of Norway in Tromsø (TMU, Norway).
Disturbance	The sedimentary cores were retrieved from the deepest part of the lakes using a floating platform. There was no disturbance in the environment.

Reporting for specific materials, systems and methods

We require information from authors about some types of materials, experimental systems and methods used in many studies. Here, indicate whether each material, system or method listed is relevant to your study. If you are not sure if a list item applies to your research, read the appropriate section before selecting a response.

Materials & experimental systems

n/a	Involved in the study
☒	☐ Antibodies
☒	☐ Eukaryotic cell lines
☒	☐ Palaeontology and archaeology
☒	☐ Animals and other organisms
☒	☐ Human research participants
☒	☐ Clinical data
☒	☐ Dual use research of concern

Methods

n/a	Involved in the study
☒	☐ ChIP-seq
☒	☐ Flow cytometry
☒	☐ MRI-based neuroimaging
